# A Sugaromics Method for Combined Targeted and Untargeted Sugar Profiling: Fit-for-Purpose Validation of a Quantitative GC × GC‑MS Approach

**DOI:** 10.1021/acs.analchem.5c04230

**Published:** 2026-03-18

**Authors:** Elisa E. Streitenberger, Björn Egert, Lara Frommherz, Sabine E. Kulling, Carina I. Mack

**Affiliations:** Department of Safety and Quality of Fruit and Vegetables, 14878 Max Rubner-Institut., Haid-und-Neu-Straße 9, 76131 Karlsruhe, Germany

## Abstract

The composition of sugar compounds in human biofluids is affected by various factors, including diet, health, demographic background, and lifestyle. Accurate quantification of this profile enables identification and application of biomarkers linked to health and nutrition, offering valuable insights into the mechanistic background of sugar metabolism. However, existing methods typically quantify only a few sugar compounds simultaneously, constraining full assessment of the sugar profile. Hence, we propose a comprehensive two-dimensional gas chromatography coupled with a mass spectrometry-based detector sugaromics method applying a nonpolar-medium polar column setup. The method is a combination of a targeted and untargeted approach to absolutely quantify various sugars in urine (*n* = 40) and serum (*n* = 36) and simultaneously identify untargeted sugar compounds in urine (*n* = 35) and serum (*n* = 22). The method was evaluated through a fit-for-purpose validation using the guidelines of the U.S. Food and Drug Administration and of the International Council for Harmonisation of Technical Requirements for Pharmaceuticals for Human Use as guiding principles. The majority of sugars demonstrated satisfactory validity parameters in terms of linearity, lower and upper limit of quantification, precision, accuracy, and carryover. Furthermore, the validated method was applied to human urine and serum samples (*n* = 40), indicating quantifiable analyte concentrations within the expected range. In this respect, for healthy adults, absolute concentrations of seven sugars in urine and 14 in serum were reported for the first time. In conclusion, the newly developed method combining targeted and untargeted approaches demonstrates good performance and is promising for application in human studies investigating health and nutrition.

## Graphical abstract

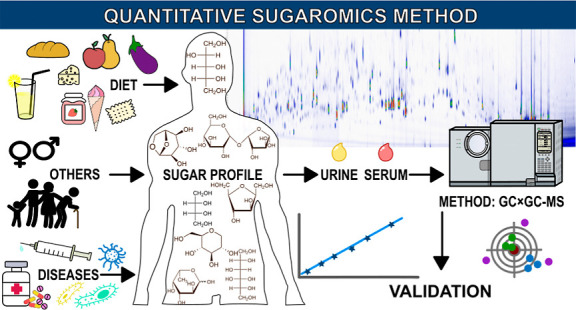

The sugar profile of human biofluids is highly complex, with up to 84 different sugar compounds detected simultaneously in urine and blood.^1–3^ In this manuscript, we use the term “sugar” or “sugar compounds” to refer to simple carbohydrates, that is, monosaccharides and lower oligosaccharides, as well as substances derived from monosaccharides through reduction, oxidation, or replacement of the carbonyl- or hydroxy-group according to the IUPAC definition.^4^ Previous literature mainly focuses on the quantification of few selected sugars such as glucose, fructose, *myo*-inositol, 1,5-anhydroglucitol, glucaric acid, sorbitol, and arabitol in biofluids (see Tables S2 and S3, Supporting Information S1), resulting in a knowledge gap regarding concentrations of other sugars in urine and serum of healthy adults. Furthermore, only a limited number of analytical methods^5–12^ quantified 10 or more sugar compounds simultaneously. This is surprising given the complexity of the sugar profile and its relevance to various factors affecting it, such as diet (e.g., total sugar,^13–15^ fruit,^16–19^ vegetables,^3^ dairy products,^3,20^ or alcohol intake^3^), diseases (e.g., diabetes mellitus,^2,7,21,22^ invasive candidiasis,^23^ uremia,^7^ myocardial infarction,^24^ some inborn errors,^6,25–30^ or cancer^5,31,32^ ), and demographic and life style factors^3,6,33,34^ (e.g., age and sex). Absolute quantification methods enable the identification of biomarkers for health or diet to improve diagnosis, prognosis, and monitoring of diseases, as well as generally improve our understanding of the metabolic pathways of various sugars.

The commonly applied methods for quantifying sugars in biofluids include high-performance liquid chromatography (HPLC), gas chromatography (GC), nuclear magnetic resonance spectroscopy (NMR), or enzymatic assays (see Tables S2 and S3, Supporting Information S1). Enzymatic assays typically quantify only 1–3 sugars, making them unsuitable for a multitargeted approach. Furthermore, NMR lacks the necessary sensitivity^35^ for quantifying most of the sugars with concentrations in the nano/micromolar range. To quantify multiple sugars without complex sample cleanup, method separation efficiency is essential, given the high structural similarity of the sugars. Among those analytical methods capable of quantifying more than 10 sugars simultaneously, GC and LC are predominantly used. Furthermore, the selection of a suitable detector plays a critical role in ensuring both the sensitivity and specificity of measurements. Mass spectrometry-based detectors (MS) are highly sensitive and enable separation of coeluting substances based on their distinct mass spectra; however, they encounter limitations when dealing with isomeric compounds. Comprehensive two-dimensional GC coupled with MS (GC × GC-MS) is especially well-suited for multitargeted sugaromics because it improves sensitivity through band focusing in the modulator and enhances separation efficiency.^36^ Mack et al.,^1^ demonstrated this by refining and extending a one-dimensional GC–MS method into a GC × GC-MS approach, which enabled the separation and relative quantification of 84 sugars (including structurally similar isomers) compared to 55 in the original method.

Within this work, a GC × GC-MS sugaromics method is presented that simultaneously enables absolute quantification of targeted sugars and relative quantification of additional sugars through an untargeted approach in human body fluids with particular emphasis on its validation. The purpose of this work was to establish a reliable method to quantify 51 sugar compounds in urine and 41 sugar compounds in serum. Sugar compounds were selected for quantification based on the availability of reference substances and after confirming that they occur regularly in the biological matrices at sufficient high signal intensity for quantification. To achieve this, the method was validated using the fit-for-purpose concept, meaning that the newly developed method meets all selected validation criteria (linearity, lower limit of quantification (LLOQ), upper limit of quantification (ULOQ), precision, accuracy, and carryover, assessed in urine and serum) required for its intended application,^37^ providing evidence that the method is suitable for the intended analytical task. To evaluate the validation, the guidelines of the U.S. Food and Drug Administration (FDA)^37^ and of the International Council for Harmonisation of Technical Requirements for Pharmaceuticals for Human Use (ICH)^38^ were used as guiding principles, even though these guidelines primarily focus on validation of a limited number of analytes, in contrast to the multitargeted method described here. In addition, a comparison with reference methods (HPLC method, enzymatic measurement) was performed for some common sugars (glucose, fructose, and sucrose). Furthermore, a comprehensive literature search was carried out in order to assess the current state of published sugar concentrations and methods for sugar quantification in the literature. Key features of the presented sugaromics method are (i) its applicability in two matrices (urine and serum), (ii) simultaneous determination of structurally similar sugar compounds across varying broad concentration ranges in biological samples using isotopically labeled internal standards (ISTD) and external calibration, and (iii) the possibility to relatively quantify additional unknown sugars using an untargeted approach. The present method including data processing workflow was adapted for the use of ISTD. Finally, the resulting validated concentration ranges are compared with concentrations found in real urine and serum samples (*n* = 40) and with the collected literature values.

## Methods and Materials

A comprehensive literature search was conducted to obtain an overview about published concentrations of sugars in urine and serum and applied methods (see Section S1, Supporting Information S1).

### Chemicals and Materials

Sugar and retention index (RI) standards as well as chemicals were acquired from different companies with purities above 95% (see Table S4, Supporting Information S1). RI standards were prepared in heptane (250 μmol/L; RI fatty acid methyl ester: C7–C26; RI alkane: C8–C30). Methoxylamine (Sigma-Aldrich, USA) was dissolved in anhydrous pyridine (dried using a molecular sieve, Carl Roth, Germany) to obtain a 20 mg/mL solution. The equipment used for sample preparation can be found in Table S5, Supporting Information S1.

### Preparation of Internal and Calibration Standards

Stock solutions of all sugar standards were made using ultrapure water. For the calibration levels (validation measurement series: urine 8 levels, serum 7 levels; application measurement series: 7 levels for each matrix), solutions for spiking (validation measurement series: 3 levels for each matrix; application measurement series: 2 for each matrix), and ISTD, the stock solutions were diluted and combined, ensuring that each sugar had its own specific concentration range (see Tables S6–S8, Supporting Information S1). An exception was the calibration curve for glucose quantification in serum, which required separate calibration levels because of the high glucose concentrations in serum. All solutions were stored at −26 °C.

### Preparation of Samples and Calibration Curve

A selection of human samples were included for method validation (urine: *n* = 3 and serum: *n* = 3) and the application study (urine and serum: *n* = 40; see section “Application of the Sugaromics Method in Urine and Serum Samples”). These samples were obtained from KarMeN and the FoodBAll study. Detailed descriptions of both studies can be found in Bub et al.^39^ and Mack,^1^ respectively. Both studies were in accordance with the 1964 Helsinki declaration and its later amendments; the local ethics committee approved both. Furthermore, quality control (QC) samples were prepared using different urine and serum samples, respectively.

Urine samples were normalized to osmolality to ensure comparable responses during measurement (osmolality values in Supporting Information S1 in Table S9). Serum samples with the addition of ISTD underwent a protein precipitation using cold methanol. In the case of urine and calibration solutions, ISTD was added in the vial. Diluted urine, serum supernatant, and calibration solutions including ISTD were then evaporated to dryness and derivatized using a two-step procedure before analysis using GC × GC-MS. For derivatization, reagents were added in excess to ensure complete derivatization and avoid competitive suppression effects. Detailed information for the sample preparation workflow is provided in Figure 1.

**Figure 1:**
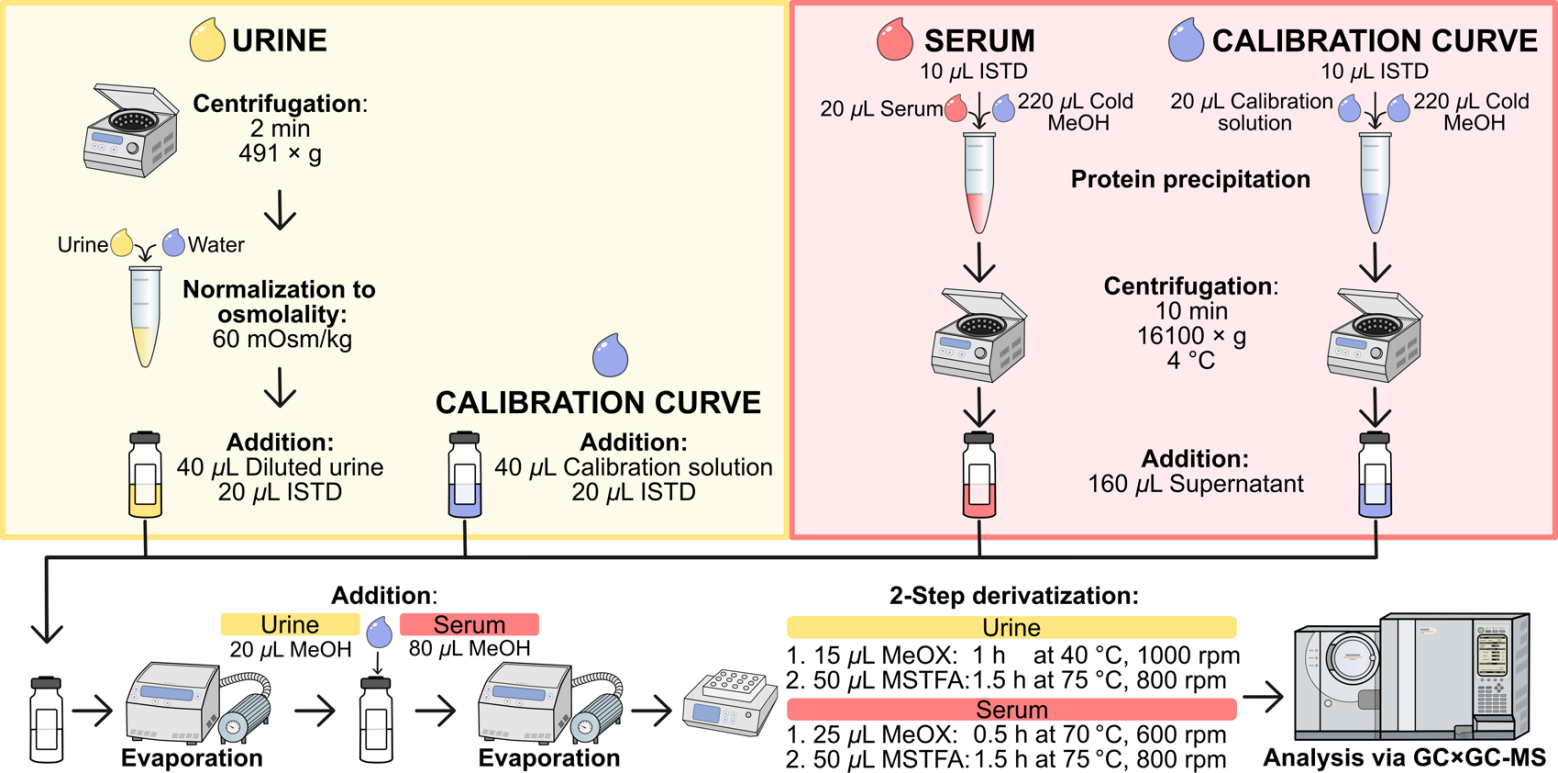
Workflow for sample preparation of urine (yellow) and serum samples (red) including their corresponding calibration curves. ISTD: isotopically labeled internal standards. MeOH: methanol. MeOX: methoxylamine. MSTFA: *N*-methyl-*N*-trimethylsilyltrifluoroacetamide.

### GC × GC-MS Analysis

GC × GC-MS analysis was performed using a GCMS QP2010 gas chromatograph coupled with a ZX-2 thermal modulator (ZOEX, USA), an OPTIC-4 PTV injector (ATAS, Netherlands), and an AOC-5000 Plus autosampler (Shimadzu, Japan). The column combination consisted of a nonpolar Rxi-5 SilMS (Restek, Germany; 40­(+5) m × 0.18 mm ID × 0.36 μm film thickness) column connected with a medium polar BPX-50 (Trajan, Australia; 2.1 m (thereof 0.9 m as modulator loop and 1 m for 2D separation) × 0.15 mm ID × 0.15 μm film thickness) column using a SilTite μ-Union column connector (Trajan, Australia) in the case of urine and in the case of serum with a medium polar V17 ms (Agilent, USA; 2.1 m (thereof 0.9 m as modulator loop and 1 m for 2D separation) × 0.15 mm ID × 0.15 μm film thickness) column. Helium (purity 5.0) was used as a carrier gas in constant linear velocity mode (urine: 25.4 cm/s; serum: 25 cm/s) at initial head pressures of 246 and 272.4 kPa for urine and serum, respectively. A volume of 1 μL was injected using a programmed temperature vaporization (90–280 °C; 60 °C/s) with a split ratio of 1:5 (after 1 min: 1:40 and after 20 min: 1:10) in the case of urine. For serum analysis, 1 μL was injected into the SPL injector at 280 °C with a split ratio of 1:5 (after 1 min: 1:50 and after 20 min: 1:10). For urine analysis, the GC temperature ramp was as follows: 80 °C → 8 °C/min → 140 °C → 1.75 °C/min → 170 °C → 1.5 °C/min → 210 °C → 8 °C/min → 255 °C → 3 °C/min → 300 °C (hold for 3.07 min), resulting in a total run time of 75 min. For serum analysis, the GC temperature ramp was as follows: 100 °C → 12 °C/min → 150 °C → 1.75 °C/min → 220 °C → 8 °C/min → 280 °C → 3 °C/min → 310 °C → 25 °C/min → 330 °C (hold for 6.53 min), resulting in a total run time of 69 min. The modulation times were 2.4 and 2.1 s for urine and serum, respectively. The hot jet temperature programming was programmed stepwise with each step of the temperature program being 100 °C above the oven temperature, starting at 200 °C. The interface temperature was set to 300 °C, while ion source temperature was held at 200 and 250 °C for urine and serum, respectively.

MS measurement was performed in scan mode with a spectral scan rate of 100 Hz, and electron ionization spectra were recorded using 70 eV. Mass ranges were changed over the time course of the run to cover the respective sugar compounds accordingly. The overall mass range covered 203 to 420 *m*/*z* with time-dependent windows (see Table S10, Supporting Information S1).

### GC × GC-qTOF Analysis

GC × GC coupled with high-resolution MS analysis was performed to support the identification of untargeted compounds in the QC samples using a 8890 GC (Agilent, USA) coupled with a quadrupole time-of-flight MS (qTOF; 7250 GC/Q-TOF; Agilent, USA), a PAL 3 RSI autosampler (CTC Analytics AG, Switzerland), and a ZX1 thermal modulator (ZOEX, USA) controlled by Optimode V.2 (SRA Instruments, Italy). Detailed GC and MS parameters are described in Table S11, Supporting Information S1.

### Data Processing

AnalyzerPro XD (version 1.16.8, SpectralWorks Ltd., U.K.) was used for automated peak integration in feature mode. The algorithm performed baseline correction, peak detection, noise reduction, and deconvolution. To reduce noise, selected masses characteristic of baseline noise were excluded for integration in the entire chromatogram, while in the disaccharide region of the chromatogram, only masses characteristic of disaccharides were utilized for integration of urine samples. Detailed integration parameters can be found in Table S12, Supporting Information S1. Per run, three csv-files were created as a result, (i) summary file with retention time (RT), features, area, height, etc.; (ii) spectra file containing nondeconvoluted apex spectra; and (iii) component file containing the feature list with the separate masses. These resulting csv-files per run for the general sugar and in the case of urine samples the additional disaccharide region of the chromatogram were imported and reorganized in a tabular structure using R-based modules (R version 4.4.2). During this step, RT was converted into ^1^D- and ^2^D-RT (RT in the first and second dimension/column) based on the modulation time, and peak heights, as well as apex spectra, were extracted. Following this, a data filtering cascade was applied to reduce the data. Noise peaks, artificial peaks, peaks below a certain threshold, nonsugar peaks (to some extent), and peaks with less than 10 neighboring peaks of a certain spectral similarity across all runs were removed. For details, see Figure S1, Supporting Information S1. Afterward, RT shifts were corrected using ISTD, which were evenly distributed across the chromatogram in four chromatographic regions. The highest modulations for each of the ISTD was annotated (comparison with in-house spectral library: minimal similarity 70% using mass set characteristic for labeled ISTD, RI tolerance ± 3, ^2^D-RT allowance ± 0.06 s) and then used to calculate the median shift within each predefined chromatographic region (see Figure S2 and Table S13, Supporting Information S1). Using the median shift minimized the impact of deviations in a single ISTD on the overall RT correction.

For alignment, modulated peaks were first clustered at the individual run level based on mass spectral similarity similar to what has been described by Egert et al.^40^ However, a reduced mass set was used to minimize the influence of ISTD and saturated peaks. Due to the high spectral similarity of sugars, modulations of two sugar compounds may sometimes be grouped into the same analyte cluster. To address this, clusters were assessed and split based on the detection of a minimum between the height of modulations of the two compounds. After this, the center of each cluster, defined as the peak from the highest modulation, was identified in all individual runs. Based on the centers of clusters over all runs, global analyte alignment was performed by aggregating the local analyte cluster center points with allowances for both ^1^D- and ^2^D-RT (xmod (^1^D-RT): ±1; yscan (^2^D-RT): ±2), resulting in a global analyte cluster over all runs. To ensure that no closely neighboring compounds were assigned in the same cluster, clusters were again assessed and, if necessary, split based on the detection of a minimum between the height of modulations of the two compounds. Finally, peaks were demodulated, meaning that all peak heights within a run assigned to the same cluster were summed, resulting in the final data matrix with runs as row and analytes as columns.

For further processing of sugar compounds for targeted quantification, identification was based on reference substances and the in-house database including >130 sugar compounds, covering various sugar isomers (MSI level I). When available, the corresponding ISTD was used with some minor exceptions; otherwise, the best-fitting standard was selected (Tables S14 and S15) based on its correlation with the sugar compound in QC samples. For example, because no ISTD was available for sedoheptulose, we used isotopically labeled mannoheptulose (structurally similar C7-sugar) as the ISTD for urine quantification. The high QC sample correlation (*r* = 0.9847) indicates efficient correction of matrix and drift effects with minimal bias. Median values were used for evaluation of validation parameters. *R*-based codes, generated with support of ChatGPT (OpenAI, 2024), were used to create Figures 2–4 in this manuscript.

**Figure 2:**
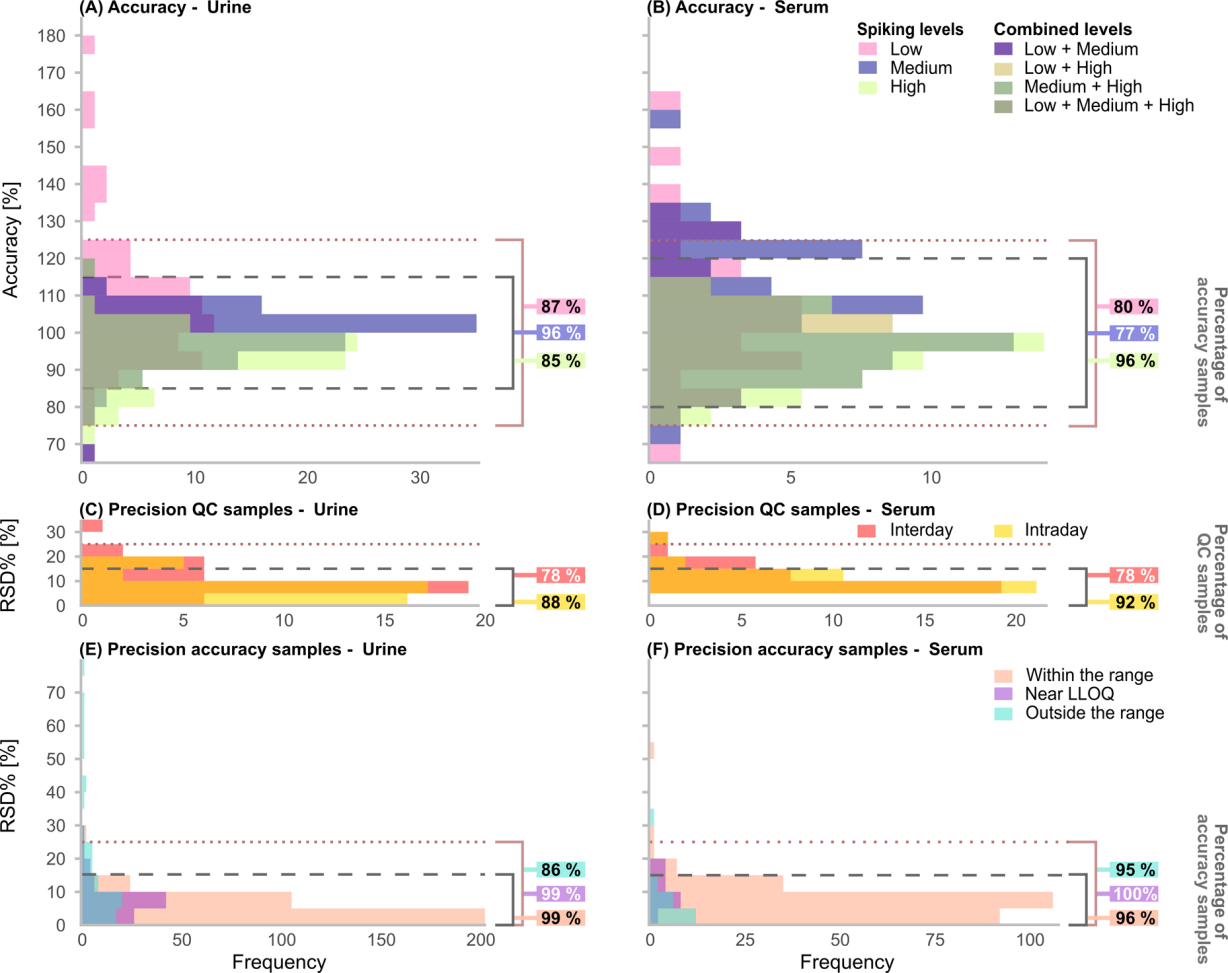
Overview of the validation parameters accuracy and precision. Percentage of accuracy/QC samples indicates how many evaluable samples in % fall within the defined limits. Dashed lines indicate the range of acceptable deviation. Histogram of panel (A) shows accuracy in urine with up to 32, 37, and 30 evaluable sugars for low, medium, and high spiking levels, respectively; panel (B): accuracy in serum with up to 22, 35, and 28 evaluable sugars for low, medium, and high spiking levels, respectively; panel (C): inter- and intraday precision of the QC samples in urine (evaluable sugars: 40); panel (D): inter- and intraday precision of the QC samples in serum (evaluable sugars: 36); panel (E): interday precision of the accuracy samples in urine (evaluable sugars: 40); and panel (F): interday precision of the accuracy samples in serum (evaluable sugars: 36). RSD%: relative standard deviation. QC: quality control. LLOQ: lower limit of quantification.

### HPLC Coupled to a Refractive Index Detector (HPLC-RI) Analysis

For the purpose of validation, several fruit juices were analyzed using HPLC-RI. Briefly, the sugar determination was carried out according to Van Den et al.^41^ and the German Reference method “Determination of individual sugars in food by HPLC” (§64 LFGB L 40.00-7)^42^ with minor modifications. Further details can be found in Section S9.1, Supporting Information S1.

### Method Validation

In total, 51 different sugars in urine and 41 in serum were validated in terms of calibration range, linearity, LLOQ, ULOQ, precision, accuracy, and carryover.

For the quantification of sugars, an external calibration using up to 31 different ISTD was applied. Each calibration curve comprised seven or eight concentration levels for serum and urine, respectively, and each level was repeated at least six times. Linearity was assessed using coefficient of determination values (*R* ^2^) and the deviation between the nominal concentration and the back-calculated concentration of each calibration standard (BCCS).^38^ LLOQ and ULOQ were defined as the lowest and highest concentration levels that could be reliably detected with acceptable precision of ≤25% and ≤15%, respectively.

For the determination of accuracy, two approaches were chosen. First, due to the lack of a blank matrix for urine or serum samples without sugar compounds, accuracy was determined by spiking samples. Therefore, three different urine and two serum samples were spiked with each sugar at three different concentration levels (*n* = 5, respectively, for low, medium, and high). In detail, 20 μL of the spiking solution was added to the urine and serum samples, respectively, before samples were further processed. The exact concentrations applied can be found in Table S6, Supporting Information S1. To determine the accuracy, the measured concentration in the unspiked sample was subtracted from the found concentration in the spiked sample and subsequently divided by the spiked concentration for each sugar. Second, in the case of some sugars, a comparison with quantification results obtained with other reference methods (enzymatic and HPLC method) was applied to determine accuracy. Due to the naturally high concentrations in serum samples, the spiking procedures were not feasible for glucose. Therefore, results obtained by our sugaromics method were compared with separate measurements in two selected serum samples: (i) one selected sample by a certified clinical chemistry laboratory (MZV Labor PD Dr. Volkmann, Karlsruhe, Germany) and (ii) the other sample using an enzymatic kit for quantification (Roche, Switzerland). To further validate the method with respect to its accuracy, sucrose, glucose, and fructose were quantified in selected fruit juices, purées, and plant drinks (apple, apricot, aronia, pear, peach, sauerkraut, and oat milk) using both the sugaromics method (see Section S9.2, Supporting Information S1) and an HPLC-RI method. Fruit juices were selected as a substitute matrix as a comparison of biofluids was not feasible owing to the high LLOQ of the HPLC method. Accuracy was evaluated by calculating the deviation between the sugar concentrations obtained by the two different methods.

Intra- and interday precision was assessed by calculating the RSD%. The QC samples were measured 10 times for intraday or 52 times for interday validation in urine and 10 times for intraday or 40 times for interday validation in serum. Furthermore, the interday precision was determined in the different accuracy samples (*n* = 5). Validation of untargeted sugar compounds included only intra- and interday precision of QC samples.

Lastly, carryover was evaluated in a separate analysis by injecting a blank sample after each calibration level (urine: 8 levels; serum: 7 levels) and repeating the entire sequence three times.

### Untargeted Analysis

Untargeted identification was classified according to MSI confidence levels (MSI level I–IV).^44^ All sugar compounds with an available reference standard were classified as MSI level I. Sugars with spectral matching ≥80% to library spectra (NIST or in-house database) and acceptable RI deviation (±30) were assigned to MSI level II. Compounds that showed (i) characteristic sugar fragment ions and/or (ii) a spectral match ≥70% but (iii) present RI deviation greater than ±30 were categorized as a subclass of sugar compounds (MSI level III). Their exact fragment ion *m*/*z* values were measured using GC × GC-qTOF analysis and compared with theoretical exact fragment ion *m*/*z* of sugar compounds to strengthen identification confidence (see Table S16, Supporting Information S1).

### Application of the Sugaromics Method in Urine and Serum Samples

In 40 urine and serum samples from the KarMeN study^39^ (for participant characteristics, see Table S17, Supporting Information S1), 38 and 35 sugar compounds, respectively, were quantified in two separate measurement series. For each series, a short validation including LLOQ, ULOQ, linearity, accuracy with two spiking level, and interday precision of QC samples and calibration levels was performed. Concentration of the calibration levels, ISTD, and spiking solution are presented in Supporting Information S1 in Tables S7 and S8. Sample preparation, GC × GC-MS analysis, and data processing were performed as described above with a few exceptions (see Table S18, Supporting Information S1).

## Results and Discussion

### Method Validation

Method validation was finally performed for 40 sugar compounds in urine and 36 in serum. Reasons for exclusion of sugar compounds from validation included chromatographic issues like coelution and trace compounds, a lack of suitable ISTD for matrix correction, and technical issues (urine: glucosamine, galactosamine, and *scyllo*-inositol; serum: glucosamine, ethyl glucoside, threonic acid, glucaric acid, and sorbitol). Lactones of sugar acids (*n* = 6) were generally excluded due to difficulties with the equilibrium between acid and lactones in the matrices and the lack of availability of the respective sugar acid reference compounds. Although these sugars could not be quantified absolutely, relative quantification was possible.

To assess linearity, LLOQ and ULOQ, an external calibration with seven or eight concentration levels repeated at least six times depending on the measurement series length for serum and urine, respectively, was applied using ISTD to correct matrix effects and errors occurring during sample preparation. Calibration levels with higher variability (RSD% > 15%, except at LLOQ > 25%) were excluded. A few exceptions were made for urine (*n* = 8 sugars) and serum (*n* = 8 sugars), allowing slightly higher RSD% at LLOQ or ULOQ (up to 6% beyond accepted limits) and for calibration levels in the middle of the calibration curve (up to 12%), where surrounding levels had acceptable RSD%. For the remaining calibration levels, the best regression model (linear, 1/*x*-weighted, 1/*x* ^2^-weighted or quadratic) was determined by evaluating *R* ^2^ and the BCCS. After exclusion of levels that (i) led to flattening of the curve due to detector saturation or (ii) showing overlapping *m*/*z* between the sugar and its isotopically labeled counterpart, all *R* ^2^ were >0.99, and the BCCS were within ±15% of the nominal value (except at LLOQ ± 25%) with minor exceptions at LLOQ.

Consequently, for each sugar, an individual calibration range (LLOQ–ULOQ) with 5–8 concentration levels (generally 6 and 7 levels in serum and urine, respectively) was established. Calibration range (LLOQ–ULOQ), selected regression model, *R* ^2^, and the deviation of the BCCS for both matrices are presented in Tables S22 and S23, Supporting Information S2.

The FDA and ICH guidelines recommend to measure at least six calibration levels, using the simplest regression model and BCCS should be within ±15% (except at LLOQ ± 20%) for 75% of the calibration levels.^37,38^ As demonstrated, the quantitative sugaromics method adheres to these guidelines, with slight deviations. Only in six cases, a more complex regression model (1/*x* ^2^-weighted, quadratic) was selected due to correctable saturation effects for the selected *m*/*z* or overlapping *m*/*z* between the sugar and its isotopically labeled counterpart. Since this multitarget sugaromics method stems from metabolomics, where RSD% of ±25% is common,^45^ we set limits at the LLOQ to ±25%. In contrast, guidelines accept ±20%, but we accepted a broader range due to the multitargeted nature of our method.

Accuracy and precision were assessed to prove reliability and trueness of the obtained concentrations. The summarized accuracy in urine and serum grouped according to low, medium, and high spiking levels is shown in Figure 2A,B and precision in Figure 2C–F. Detailed information on accuracy and precision for each sugar compound is presented in Tables S22 and S23, Supporting Information S2. In general, more accurate values outside of the accepted range were observed in serum samples compared to urine, possibly due to the more complex sample preparation including protein precipitation.

To examine the accuracy of different spiking levels, note that measurements for some sugar compounds could not be evaluated for one or more sample and spiking levels due to (i) measured signals falling outside the previously determined calibration range or (ii) spiked amounts being less than 25% of the concentration in the unspiked sample. Furthermore, for three sugars in urine (rhamnose, galacturonic acid, and *N*-acetylglucosamine/-mannosamine), accuracy could not be evaluated as the unspiked sample’s signal was below LLOQ. Nevertheless, for those four sugars, borderline to acceptable accuracy (low: 79–122%, medium: 75–112%; and high: 86–113%) could be achieved based on the extrapolation of the calibration curve for concentration determination in the unspiked sample. This suggests that the linear calibration range might extend beyond what was determined by the precision of the lowest calibration levels and the linearity of the calibration curve.

Compared to FDA and ICH guidelines,^37,38^ the majority of evaluable accuracy (91% in urine and 76% in serum for medium and high levels) was within the accepted 85–115% range. In serum, due to complex sample preparation, we accepted a wider range (80–120%), with 85% of the values falling within this range. At the lowest spiking level, we set accuracy limits to 75–125% and precision limits for samples near the LLOQ to RSD% ≤ 25% due to the multitargeted nature of our sugaromics approach, in contrast to the guidelines (accuracy: 80–120% and RSD% ≤ 20%). 87% of the determined accuracy in urine and 80% in serum fell within this range. Generally, accuracy outside the accepted range was observed when detector saturation or spiked amounts being too low relative to the sample matrix occurred, a less suitable ISTD was used for correction (threitol in serum: accuracy <85%), or closely coeluting substances were present (trehalose accuracy in serum >120%).

Considering the intraday and interday precision of the QC and accuracy samples, the majority of the determined RSD% were below 15% and 25% at LLOQ, respectively (see Figure 2C–F).

The accuracy of glucose in serum was not determined by spiking due to the high natural concentration of glucose; instead, accuracy was determined by comparison with enzymatic reference methods. Both measured serum samples exhibited acceptable deviation from the reference method ≤8% (serum 1: 4.56 mmol/L vs reference value 4.41 mmol/L and serum 3: 5.09 mmol/L vs reference value 4.70 mmol/L) and lay in the concentration range for glucose in the literature (2.66–8.40 mmol/L, see Table 1 and Table S3, Supporting Information S1), proving the applicability of the GC × GC-MS sugaromics method.

**Table 1:** Measured Sugar Concentrations in Urine (Normalized to Creatinine Excretion, to 24 h Urinary Volume, or Not Normalized) and Serum Samples^e^

	urine				serum	
sugar compound^a^	*n* ^b^	μmol/mmol creatinine^c^	μmol/24 h^c^	μmol/L^c^	*n* ^b^	μmol/L^c^
1,5-AG	40	1.4 ± 0.7	17.2 ± 7.9	8.1 ± 7.3	40	115.0 ± 36.4
2,4-DHB	40	13.3 ± 18.8	148.5 ± 147.1	69.8 ± 81.9	40	0.7 ± 0.3
allose		nq	nq	nq	40	0.4 ± 0.3
arabinonic acid		nq	nq	nq	40	1.1 ± 0.6
arabinose	40	21.3 ± 11.8	256.2 ± 121.8	119.1 ± 92.7	40	1.5 ± 0.4
arabitol	40	32.6 ± 14.3	398.1 ± 176.9	183.7 ± 134	40	2.9 ± 1.0
*chiro*-inositol	39	1.3 ± 2.0	13.5 ± 20.3	6.4 ± 11.8		nq
erythritol	40	77.8 ± 127	1082.6 ± 2628.1	483.6 ± 1059.7	40	5.3 ± 7.4
erythronic acid		nq	nq	nq	40	4.7 ± 1.3
erythrose		nd	nd	nd		nq
fructose	39	14.7 ± 16.8	165.8 ± 151.3	79.7 ± 90.8	40	9.2 ± 1.4
fucitol	40	0.6 ± 0.2	7.3 ± 2.8	3.5 ± 2.9		nq
fucose	40	9.2 ± 4.6	110.0 ± 50.9	53.5 ± 45.6	40	1.0 ± 0.5
galactitol	40	5.4 ± 6.9	62.5 ± 58.6	28.8 ± 32.2	40	0.4 ± 0.4
galactose	38	6.8 ± 9.4	83.2 ± 103.5	36.1 ± 41.0	40	0.2 ± 0.1
galacturonic acid	40	0.3 ± 0.3	4.3 ± 4.2	2.1 ± 2.8		nq
glucaric acid		nd	nd	nd		nq
gluconic acid	39	25.3 ± 15.4	291.7 ± 117.9	133.5 ± 93.9	40	2.2 ± 0.6
glucose	40	27.7 ± 13.2	328.9 ± 120.0	156.5 ± 114.7		4.8 ± 0.4^d^
glucuronic acid	40	27.9 ± 11.7	332.6 ± 109.7	152.9 ± 94.1	40	4.0 ± 1.0
lactose	40	6.9 ± 8.2	78.5 ± 76.2	38.8 ± 51.8	40	0.3 ± 0.1
levoglucosan	40	4.9 ± 4.4	63.7 ± 62.6	26.3 ± 26.9	35	0.3 ± 0.5
maltose	32	0.5 ± 1.2	4.9 ± 10.4	1.8 ± 3.1	40	0.5 ± 0.1
mannitol	39	123.1 ± 191.1	1521.5 ± 2606.1	655.0 ± 1068.6	40	3.1 ± 3.5
mannoheptulose	39	2.6 ± 1.6	32.3 ± 23.5	16.1 ± 20.8	40	0.2 ± 0.9
mannose	40	1.4 ± 1.8	16.6 ± 20.3	7.3 ± 7.3	40	59.8 ± 7.9
*myo*-inositol	40	13.6 ± 14.4	151.8 ± 134.4	70.2 ± 85.7	40	27.9 ± 4.4
psicose	39	62.7 ± 194.7	638.8 ± 1536.0	326.0 ± 813.0	40	2.1 ± 3.0
rhamnose	40	0.9 ± 0.5	10.6 ± 6.7	5.1 ± 5.3		nq
ribitol	40	4.4 ± 2.1	53.2 ± 23.0	24.5 ± 18.4	40	0.4 ± 0.1
ribonic acid		nq	nq	nq	40	0.3 ± 0.1
ribose	40	5.2 ± 1.9	64.1 ± 20.6	29.9 ± 20.5	40	1.7 ± 0.6
*scyllo*-inositol		nq	nq	nq	40	1.3 ± 0.6
sedoheptulose	40	8.2 ± 4.0	101.6 ± 56.4	52.0 ± 56.7	40	0.8 ± 0.3
sorbitol	40	6.2 ± 5.0	75.3 ± 55.0	34.6 ± 31.5		nq
sucrose	40	5.9 ± 6.8	66.3 ± 60.3	31.1 ± 32.7	38	0.6 ± 0.6
tagatose	38	2.2 ± 5.9	22.5 ± 48.3	10.5 ± 23.9		nq
threitol	40	26.8 ± 26.8	308.6 ± 234.9	141.9 ± 130.1	40	1.6 ± 1.0
threonic acid	40	21.1 ± 19.0	242.3 ± 149	110.5 ± 91.4		nq
trehalose	40	0.2 ± 0.1	1.9 ± 1.4	0.8 ± 0.7	40	0.1 ± 0.05
xylitol	40	5.3 ± 4.7	69.2 ± 73.2	29 ± 28.3	40	0.3 ± 0.1
xylonic acid		nq	nq	nq	39	0.7 ± 0.4
xylose	40	28.4 ± 20.1	342.5 ± 232.2	166.8 ± 155.2	40	0.9 ± 1.0
xylulose	40	6.3 ± 2.8	75.4 ± 27.4	34.2 ± 22.9	40	0.5 ± 0.1

a Generally, all sugar compounds are trimethylsilylated derivatives, and in the case of reducing sugars, methoximated. However, for reasons of readability, the underivatized names of the sugar compounds are used. The quantification of reducing sugars was carried out using one derivative, details on which was used are shown in Tables S26 and S27, Supporting Information S2.

b Number of samples for which quantification was possible.

c Mean ± standard deviation.

d Concentration in mmol/L.

e 1,5-AG: 1,5-anhydroglucitol; 2,4-DHB: 2,4-dihydroxybutyric acid; nq: not quantified; nd: not determinable as low linearity or high RSD% of the calibration levels were given.

To further prove the accuracy of the sugaromics method, sucrose, glucose, and fructose were quantified in selected fruit juices (*n* = 5), purées (*n* = 2), and plant drinks (*n* = 1) using both the GC × GC-MS and an HPLC-RI method as reference. Comparison of human biofluids was not possible due to the high LLOQ of the HPLC method. Figure 3 shows the percentage deviations of the three sugar concentrations from the reference method. Only fructose in sauerkraut juice and oat milk, and sucrose in apple juice showed deviations higher than 23% (see Table S20, Supporting Information S1). This can be explained by the low sugar concentrations in these drinks, which are close to the LLOQ of the HPLC method. This may lead to higher inaccuracy of the HPLC results.

**Figure 3:**
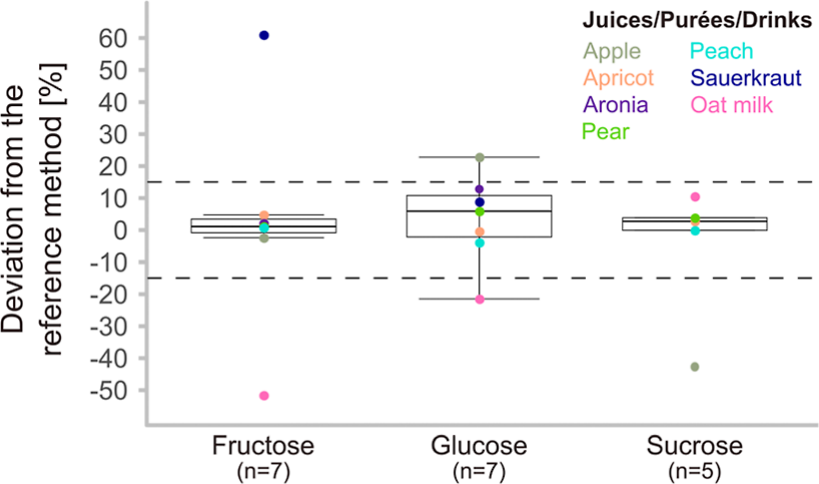
Percentage deviation of the measured values by GC × GC-MS compared to the HPLC reference method. Each data point represents the deviation of the concentration in one sample compared to the corresponding value from the reference method. Positive values indicate higher concentrations found in the newly validated sugaromics method. Dashed lines indicate the range of acceptable deviation of ±15%.

To assess carryover, blank samples were measured after the concentration levels. In general, no carryover was observed according to the ICH guideline.^38^ However, a few sugar compounds (levoglucosan in blanks after urine calibration levels; fructose, sucrose, and levoglucosan in blanks after serum calibration levels) showed sporadic peaks in blank samples, occurring independently of the concentration level measured immediately prior to the blank (see Table S21, Supporting Information S1). The impact on the quantification of sucrose in serum is negligible due to the sporadic signal in blank samples being less than 13% of the response at LLOQ. In contrast, the quantification of fructose and levoglucosan in serum near LLOQ and the next higher calibration level, as well as levoglucosan in urine at the LLOQ, is susceptible to a positive bias. This bias arises from mean residual peak heights in the blanks that exceed 20% of the peak area of the corresponding calibration level. It may lead to an overestimation of the true concentrations, so values near the LLOQ and the next higher calibration level must be interpreted with care.

### Untargeted Approach

Complementary to the targeted quantitative analysis, the sugaromics method facilitates the untargeted identification of additional sugars. To support the identification, a urinary and serum QC sample each was measured separately using GC × GC-qTOF. Even with low-energy electron ionization (12–20 eV), molecular ions could not be observed for sugar compounds, as described by Petersson.^46^ Predominantly, sugar-characteristic fragment ions (e.g., sugar acids, deoxy sugars, or polyhydroxy derivatives) were measured and their exact masses matched to theoretical sugar fragments (see Tables S24 and S25, Supporting Information S2) to assign unknowns to a sugar subclass when possible. Overall, a total of 35 and 22 sugar compounds in urine and serum, respectively, were detected in addition with acceptable intra- and interday precision (urine RSD% ≤ 20% and serum RSD% ≤ 34%). In urine, 16 sugars were identified at MSI level I and 19 at MSI level III; in serum, 11 compounds were identified each at MSI level I and III. All sugar compounds, their *m*/*z* used for identification, RI, library comparison, intra- and interday precision, MSI level, and the applied ISTD are listed in Tables S24 and S25, Supporting Information S2. Future work could expand the targeted sugaromics method to quantify further MSI level I sugars with available reference standards, such as mannonic acid or *neo*-inositol.

### Application of the Sugaromics Method

In separate measurement series, concentrations of 36 sugars in urine and 35 in serum (each *n* = 40 participants) were determined using the newly validated GC × GC-MS method. An additional short validation was performed during each measurement series to ensure accuracy, linearity, and precision (for detailed results, see Tables S26 and S27, Supporting Information S2). The analyzed subset exhibits a roughly balanced gender composition (Table S17, Supporting Information S1), and volunteers were evenly distributed across the 18–80 year range, with at least three participants in each 10 year age interval (Figure S3, Supporting Information S1). Therefore, findings can be regarded as sufficiently generalizable to a broader healthy adult population.

Figure 4 and Table 1 present all determined concentrations, the applied calibration range, and a comparison with concentrations collected through a comprehensive literature search. Osmolality was selected to normalize urine samples during sample preparation, reducing dilution-related variation and effects on derivatization during sugaromics measurement itself. Nevertheless, urinary concentrations were also calculated using the common normalization methods as represented in the literature (creatinine and 24 h excretion) and reported on an osmolality basis for comparison with the validated concentration ranges.

**Figure 4:**
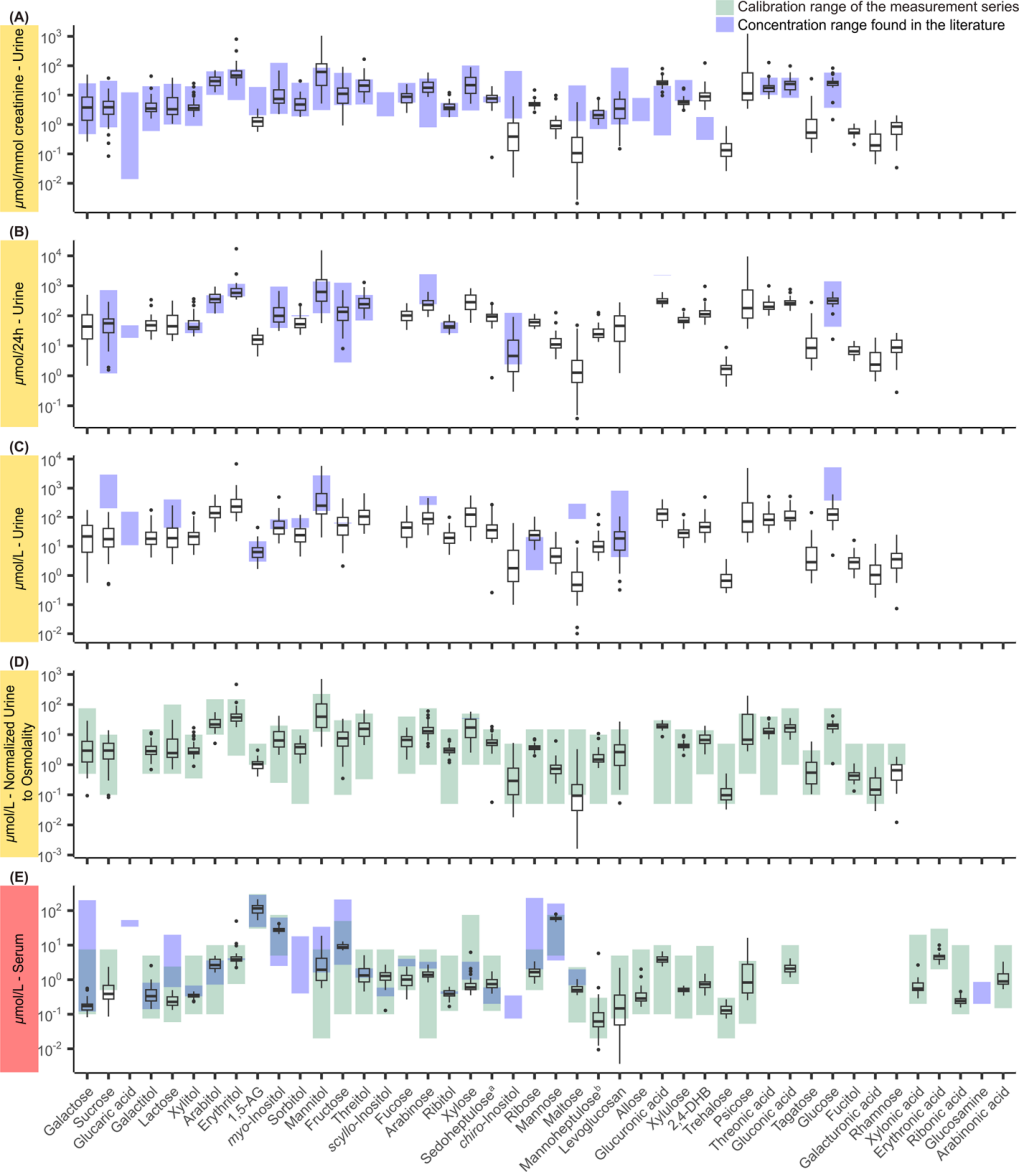
Boxplots of measured concentrations of various sugars in urine and serum (*n* = 40). The horizontal line represents the median, and the box spans the interquartile range (25–75%). Whiskers extend to 1.5× the interquartile range, with points beyond that representing outliers. The scale is logarithmic. Blue bars represent the range of the literature values that were collected through a comprehensive literature search (associated references see Tables S2 and S3, Supporting Information S1). Green bars represent the assessed calibration range of the measurement series (see Tables S26 and S27, Supporting Information S2). (A) Urine normalized to creatinine; (B) urine normalized to 24 h urine volume; (C) urine not normalized; (D) urine normalized to 56 mOsm/kg; and (E) serum. ^a^Urine literature values refer to individuals aged 0–75 years, and serum literature values refer to individuals aged 0–17 years as no separate values for adults were reported in the literature. ^b^Urine literature values refer to individuals aged 0–75 years as no separate values for adults were reported in the literature. 1,5-AG: 1,5-anhydroglucitol. 2,4-DHB: 2,4-dihydroxybutyric acid.

The predominantly applied normalization to creatinine is critical as creatinine excretion varies depending on age, sex, race, diet, physiological conditions, level of exercise, or time of day.^47–51^ In this study, normalization to creatinine and osmolality showed a strong agreement, confirmed by a robust correlation between both (*r* = 0.862, see Figure S4, Supporting Information S1), as reported in the literature.^52^ The normalization to 24 h urine volume enables us to assess total daily excretion of substances; therefore, it can be used as proxy for daily dietary intake. The normalized concentrations obtained from the 40 urine and serum samples were first compared against values reported in the literature, demonstrating overall agreement for the majority of sugars with a few exceptions where we measured lower (e.g., urine: 1,5-anhydroglucitol, *chiro*-inositol, serum: lactose, xylose) or higher (e.g., urine: 2,4-dihydroxybutyric acid; serum: *scyllo*-inositol, sedoheptulose) concentrations (Figure 4A–C,E). Next, each quantified compound in the application study was examined relative to the calibration range employed for the targeted sugaromics method; all but a few analytes (urine: 1,5-anhydroglucitol, maltose, and rhamnose; serum: levoglucosan and sucrose) fell comfortably within these limits, confirming the method’s suitability for routine quantification (Figure 4D,E). Finally, for serum, a direct comparison between the literature concentration ranges and the method’s calibration ranges revealed that, for most sugars, the existing calibration spans the physiologically relevant levels observed in healthy adults. Only a few sugars in serum (e.g., galactose, lactose, and ribose) exceed the current calibration bounds, indicating that the calibration range should be broadened for these analytes in future work. Some compounds, such as *N*-acetylglucosamine in both matrices as well as xylonic and ribonic acid in urine, were quantified as sum parameters in the validation study due to coelution. The clinical interpretability of these is limited. Consequently, the sum parameters were excluded from comparison in the application study.

Comparing the collected literature (see Tables S2 and S3, Supporting Information S1) with our results shows that absolute concentrations for seven urinary and 14 serum sugars in healthy adults are reported for the first time. In previous works, some of these sugars were associated with the diet (e.g., mannitol and mushrooms, and mannoheptulose and avocado)^3^ or health status (elevated plasma trehalose in prediabetic and diabetic volunteers vs healthy volunteers).^2^ Also, concentrations of psicose (also called allulose), a potential sugar substitute, were not reported in the literature, although we found similar and even higher concentrations in urine compared to fructose (psicose: 37.0–9538.9 μmol/24 h vs fructose: 8.1–714.2 μmol/24 h). Psicose has been reported to occur naturally in foods,^53^ especially in high-sugar foods processed at high temperatures, and is also discussed as a potential honey-adulteration marker.^54^ Therefore, psicose could potentially act as a biomarker for the intake of such foods or for food-authenticity assessment. In addition, we quantified allose in all serum samples, which is, similar to psicose, discussed to be a potential sucrose substitute due to its low calories and its physiological functions.^55^ Nevertheless, the natural occurrence of these sugars in biofluids and foods is reported seldomly. Zhang et al.^56^ reports that allose occurs in the serum of women and suggested it might act as a potential biomarker for the complementary diagnosis of hyperandrogenism and insulin resistance in polycystic ovary syndrome. Given the numerous and often conflicting associations reported in the literature for various sugars and diseases or conditions, a coherent interpretation of biological implications remains difficult. The metabolic fate of many atypical or low-abundance sugars is still insufficiently understood, further limiting mechanistic insight. The development and validation of our sugaromic method capable of quantifying such low-abundance and structurally diverse sugars will be a key step toward advancing our understanding through future studies that address these knowledge gaps.

### Strengths, Limitations, Future Directions, and Conclusion

One of the key advantages of the sugaromics method is the multitargeted nature of the approach, focusing on the substance class of sugars. This enables the selection of individual concentration ranges for each sugar in different matrices, as described in the literature.^5,6,11^ Although individual concentration ranges cause additional effort, it is necessary due to the various and broad calibration ranges in real samples (Figure 4). While we have tried to include all sugars in one calibration, this was not feasible for glucose in serum due to the larger quantities required compared to other sugars (millimolar instead of micromolar). Even though the glucose standard was of high purity (>99.5%), the applied large quantities can lead to a falsification of the calibration curves for other sugars due to trace impurities (e.g., fructose). Also, despite the fact high glucose concentrations might impair the efficiency of derivatization for low abundance sugars via competitive suppression effects, the good accuracy observed for most of the sugar compounds supports that application of derivatization reagent in abundance leads to negligible competitive suppression effects under the chosen derivatization conditions.

As shown by the comparison with literature values in Figure 4, the selected individual concentration ranges fit nicely with expected values; nonetheless, for investigation of specific scientific questions, these ranges might need to be adjusted. For instance, in postprandial samples,^13,20^ or in samples from pregnant women,^57,58^ children,^6^ or patients with certain medical conditions,^21,24^ sugar concentrations can deviate significantly from the expected ranges. The adjustment of concentration ranges should be simple and feasible; however, we recommend always performing a short validation with respect to concentration ranges or to include accuracy samples in each measurement series.

Focusing on a specific substance class provides an improved ability to account for challenges such as matrix effects, for example, by applying dedicated isotopically labeled sugar standards as ISTD. Nonetheless, it has to be mentioned that for those sugars with no matching labeled counterpart, some limitations in correction of matrix effect, and consequently quantification, have to be accepted. For instance, the multiomics method by González-Domínguez et al.^10^ with more than 600 analytes was not able to use individual isotopically labeled standards for each substance; hence, the quantification of some sugars in plasma was not possible due to matrix effects.

The fit-for-purpose validation of the sugaromics method proved the reliability of the method. Although the FDA and ICH guidelines are intended for validation of quantitative methods for only one or few substances, our method meets the necessary requirements for the parameter’s linearity, LLOQ, ULOQ, precision, accuracy, and carryover for most sugar compounds. Serum, in particular, showed larger deviations in accuracy; however, RSD% was acceptable. A possible explanation could be the more complex sample preparation applying MeOH for protein precipitation. Partial precipitation of some sugars may have contributed to the observed deviation. Future work could improve accuracy by evaluating alternative precipitation methods such as using acetonitrile or sulfate salts. Other approaches measuring multiple sugars in the literature demonstrated comparable validation results with good linearity (*R* ^2^ > 0.99), precision (RSD% < 18%), and accuracy (55–122%).^5,6,10,11^ Further methods measuring multiple sugars in the literature did not report their validation results precluding comparison.^7,9,12^ In addition to the comparison with guidelines and the literature, another strength of our approach is the additionally performed comparison with reference methods (enzymatic and HPLC) for glucose, fructose, and sucrose to confirm accuracy and reliability of the sugaromics method. Moreover, we provide validation data for two matrices, at the same time demonstrating that the approach could be quickly applied to further matrices. This highlights the potential application of the sugaromics method to other biofluids, such as feces, saliva, breast milk, cerebrospinal fluid, or even food matrices, in subsequent studies.

The combination of targeted and untargeted analysis demonstrated and confirmed previous work,^1–3^ indicating there are still more sugars present in biofluids than those quantified by the sugaromics method. In the future, it should be investigated whether additional sugars, such as phosphate sugars, deoxy sugars, and further sugar acids, can be incorporated into the quantitative approach. Moreover, to enable confident identification of untargeted compounds, future work should focus on acquiring additional reference standards.

Additionally, an extensive overview of the concentration ranges in urine and blood was provided with the help of a comprehensive literature search, showing also good agreement with measured values. Bouatra et al.^12^ likewise compared their results to literature values, but referenced only the Urine Metabolome Database without indicating the specific publications behind the reference values. Furthermore, for healthy adults, we present for the first time concentrations for seven sugars in urine and 14 sugars in serum as revealed by our literature review, thereby establishing a baseline for comparative analyses in scientific investigations.

The biggest challenge in validation is the lack of blank matrices due to naturally occurring sugars in biofluids. This makes the assessment of calibration curves, accuracy, selectivity, recovery, and limit of detection cumbersome, especially if the spiked amounts of the sugar were lower than its natural content in the samples. Identifying samples with low sugar contents that could be spiked with small amounts while still falling within the validated calibration range constituted a major constraint in this work. Hence, the determination of the accuracy at lower levels has limitations in terms of certainty; therefore, quantitative values in this range should be interpreted with caution. Furthermore, the absence of blank matrices prevented us from evaluating selectivity and recovery, which are mandatory for full FDA/ICH method validation.^37,38^ In future work, specificity, matrix effects, dilution linearity, reinjection reproducibility, and stability could be evaluated to approach full validation, although selectivity and recovery would still require additional investigation due to missing blank matrices. Further limitations with respect to the measurement itself were the saturation of the detector for selected *m*/*z* in the case of high amounts, which results in a flattening of the calibration curve, and overlapping *m*/*z* between the sugar and its isotopically labeled counterpart, particularly if one significantly predominates. Potential solutions to these problems are using other *m*/*z* for quantification, increasing or decreasing the detector voltage during the measurement in specific time ranges or choosing another ISTD for correction, if the normal sugar predominates. Furthermore, it could be considered to choose another regression model or application of two separate calibration regressions for the lower and higher concentration ranges, enabling a larger overall concentration range. In addition, quantification of some sugar compounds (xylonic and ribonic acid in urine, *N-*acetylglucosamine, and *N*-acetylmannosamine) was impeded by coelution. In the case of the quantification in serum, we successfully separated ribonic and xylonic acid by modifications of the setup. Future work should target their separation in urine and of other coeluting sugar compounds in both matrices.

In conclusion, the validated method enables accurate quantification of 40 and 36 sugars (15/13 monosaccharides, 4/4 disaccharides, 7/7 sugar acids, 12/10 polyols, and 2/2 other sugars) in urine and serum, respectively, with overall acceptable linearity, precision, accuracy, and carryover. Moreover, an application study employing 40 urine and serum samples allowed the quantification and description of physiological concentrations for 36 and 35 sugars, respectively. Additionally, the combined targeted and untargeted approach of our sugaromics method enabled additional identification and relative quantification of 35 and 22 sugars in urine and blood. Consequently, this paves the way for application of the sugaromics method to investigate human nutrition and health and to assess metabolic pathways of sugars in the future.

## Supplementary Material





## Data Availability

The data supporting the validation study are openly available in OpenAgrar: DOI: 10.25826/Data20251023-093808-0. The data supporting the application study are openly available with an embargo of 1.5 years in OpenAgrar: DOI: 10.25826/Data20251023-094945-0. The data processing scripts (R code) are available upon request.

## Notes

Detailed description and results of the comprehensive literature research; additional experimental details, materials and methods; and detailed results of the comparison with the HPLC reference method (PDF)
Detailed summary of the validation parameters for each sugar in urine and serum and results of the untargeted approach and the application study (XLSX)

## Abbreviations

1,5-AG: 1,5-anhydroglucitol
2,4-DHB: 2,4-dihydroxybutyric acid
MeOH: methanol
MeOX: methoxylamine
MSTFA: *N*-methyl-*N*-trimethylsilyltrifluoroacetamide
GC: gas chromatography
MS: mass spectrometry-based detector
GC × GC-MS: comprehensive two-dimensional GC coupled with MS
qTOF: quadrupole time-of-flight mass spectrometry
LC: liquid chromatography
HPLC: high performance LC
HPLC-RI: HPLC coupled with a refractive index detector
NMR: nuclear magnetic resonance spectroscopy
ICH: International Council for Harmonisation of Technical Requirements for Pharmaceuticals for Human Use
FDA: U.S. Food and Drug Administration
BCCS: back-calculated concentration of each calibration standard
LLOQ: lower limit of quantification
ULOQ: upper limit of quantification
QC: quality control
*R* ^2^: coefficient of determination
RSD%: relative standard deviation
RI: retention index
RT: retention time
ISTD: isotopically labeled internal standards
MRI: Max Rubner-Institut
