# Supplementary material for: A Sugaromics Method for Combined Targeted and Untargeted Sugar Profiling: Fit-for-Purpose Validation of a Quantitative GC × GC‑MS Approach
Source: Anal Chem. 2026 Mar 18;98(12):8851–62. doi: 10.1021/acs.analchem.5c04230 (PMC13044882; doi:10.1021/acs.analchem.5c04230)
Supplement: Supplementary file 1 [file ac5c04230_si_001.pdf]

Supporting Information 1

A sugaromics method for combined targeted and untargeted sugar profiling: Fit-for-purpose validation of a quantitative GC×GC-MS approach

*Elisa E Streitenberger[a], Björn Egert[a], Lara Frommherz[a], Sabine E Kulling[a] and Carina I Mack[a]**

[a] Max Rubner-Institut, Department of Safety and Quality of Fruit and Vegetables, Haid-und-Neu-Straße 9, 76131 Karlsruhe, Germany

* Email: carina.mack@mri.bund.de

TABLE OF CONTENT

S1 Literature research S5

S2 Chemicals and materials S13

S3 Calibration and internal standards S15

S4 Osmolality of urine samples S19

S5 GC×GC-MS and GC×GC-qTOF analysis S20

S6 Data processing S21

S7 Fragment ions S26

S8 Application of the sugaromics method S28

S9 Comparison with reference method S29

S9.1 High performance liquid chromatography coupled with a refractive index detector (HPLC-RI) method S29

S9.2 Sample preparation of fruit juice, purée and oat drink for sugaromics analysis S30

S9.3 Results of the analysis using the reference and sugaromics method S31

S10 Carryover S31

S11 Correlation between osmolality and creatinine S32

References S32

LIST OF TABLES

Table S1. Sugar compounds and their synonyms used for the search string. S6
Table S2. Concentrations in the literature for various sugars in urine. S7
Table S3. Concentrations in the literature for various sugars in blood, serum or plasma S10
Table S4. List of the applied chemicals and reference standards including information on purity and supplier. S13
Table S5. List of used equipment including information on supplier and application. S14
Table S6. Concentration of each sugar in the different calibration levels and spiking solutions [μmol/L] for the validation measurement series. S15
Table S7. Concentration of each sugar in the different calibration levels and spiking solutions [μmol/L] for the application measurement series. S17
Table S8. Concentration of the ISTD [μmol/L] for the quantification of urine and serum for the validation and application measurement series S19
Table S9. Osmolality of urine samples. S19
Table S10. Mass (*m/z*) and time range parameters for MS of urine and serum. S20
Table S11. GC and MS parameter for the GC×GC-qTOF analysis. S20
Table S12. Parameters for the integration method for urine and serum using AnalyzerPro XD. The integration of the urine measurements was divided into two parts, from 7-66.2 min and 66.2-74.99 min. S21
Table S13. ISTD used for correction for RT-shift in the respective time segments. S23
Table S14. Quantified sugar compounds in urine, their corresponding RI, as well as selected quantification ion, selected ISTD, and their corresponding quantification ion for the ISTD. S24
Table S15. Quantified sugar compounds in serum, their corresponding RI, as well as selected quantification ion, selected ISTD, and their corresponding quantification ion for the ISTD. S25
Table S16. Selected known characteristic fragment ions occurring in spectra of trimethylsilylated sugar compounds. S26
Table S17. Participant characteristics of the 40 KarMeN volunteers. S28
Table S18. Measurement series parameters and conditions for the 40 analyzed urine and serum samples. S29
Table S19. Accuracy for the HPLC-Method. S30
Table S20. Measured and rounded concentration of fructose, glucose and sucrose in fruit juices and plant drinks using the reference (HPLC-RI) and sugaromics (GC×GC-MS) method, as well as the percentage deviation of the measured values. S31
Table S21. Evaluation of the carryover measurements. Measured mean heights in the blank samples (n=3) after injection of a calibration level. S31

LIST OF FIGURES

Figure S1. Exemplary data filtering cascade for one urinary QC sample. S21

Figure S2. Comparison of the retention times before and after correction in the QC samples for four sugars in the different time segments. A point represents a modulation and the blue point represents the highest modulation. S22

Figure S3. Age distribution of the 40 KarMeN volunteers. S28

Figure S4. Correlation between osmolality and creatinine in 40 urine samples of the application study. S32

S1 Literature research

In this article, the quantification of different sugar compounds in urine and serum by GC×GC-MS were validated. Existing research was assessed to compare the GC×GC-MS method and measured concentrations with established methods and reported values. For each sugar compound, except glucose, the literature search was conducted individually in the database Web of Science using the following combinations of grouped search terms: (concentration* OR quantification* OR quantify OR quantitative OR amount) AND (urine OR urinary OR serum OR plasma OR blood) AND (human* OR men OR women OR patient* OR volunteer* OR participant* OR male OR female) NOT (animal* OR rat OR mouse OR mice OR pig* OR rats OR bird* OR horse* OR rabbit* OR immunoglobulin* OR *protein* OR *conjugat* OR intervention* OR *RNA OR *DNA OR sugar-sweetened beverage* OR semen OR seminal OR *antigen* OR polysaccharide* OR oligosaccharide* OR *lectin* OR *protein* OR cell* OR "in-vitro")), together with the name of the sugar compound and its synonyms (i.e. “psicose” OR “allulose”; see Supporting Information 1 Table S1). The obtained publications were filtered by title and abstract. Publications that only dealt with non-healthy subjects or in which the sugar was administered like in gut permeability tests were excluded. Also, publications presenting concentrations only in figures were excluded. Promising publications were further assessed, and relevant concentrations and methods were documented. Additional publications were found through reference lists of reviews and selected publications. Overall, for 33 sugars in urine and 26 sugars in blood/serum/plasma concentrations were found for healthy adults (see Supporting Information 1 Table S2-3).

Table S1. Sugar compounds and their synonyms used for the search string.

Sugar term	Sugar term
"1,5-anhydroglucitol" OR "1,5-anhydrosorbitol"	"glucosamine"
"1,6-anhydro-beta-D-glucose" OR "leucoglucosan" OR "levoglucosan"	"glucuronic acid"
"2,4-dihydroxybutanoic acid" OR "2,4-dihydroxy butyric acid"	"lactose"
"2-deoxyribonic acid-1,4-lactone" OR "2-deoxy-D-ribonic acid-1,4-lactone"	"maltose"
"5-deoxyarabonic acid-1,4-lactone" OR "5-deoxy-L-arabinono-1,4-lactone"	"mannitol"
"mannonic acid-1,4-lactone"	"mannoheptulose"
"*scyllo*-Inositol" OR "*scy*-inositol" OR "scyllitol"	"mannose"
"allitol"	"*myo*-Inositol"
"allose"	"*N*-acetylgalactosamine"
"arabino-1,4-lactone"	"*N*-acetylglucosamine"
"arabinonic acid" OR "arabic acid" OR "arabinonate" OR "arabonic acid"	"*N*-acetylmannosamine"
"arabinose"	"psicose" OR "allulose"
"arabitol" OR "arabinitol"	"rhamnose"
"*chiro*-inositol"	"ribitol" OR "adonitol"
"erythritol" OR "erythrit"	"ribonic acid" OR "ribonate"
"erythronic acid" OR "erythronate"	"ribono-1,4-lactone"
"erythronolactone"	"ribose"
"erythrose"	"sedoheptulose"
"ethyl glucoside" OR "ethyl D-glucoside"	"sorbitol" OR "glucitol"
"fructose"	"sucrose" OR "saccharose"
"fucitol"	"tagatose"
"fucose"	"threitol" OR "threit"
"galactitol" OR "dulcitol" OR "dulcit"	"threonic acid" OR "threonate"
"galactosamine"	"trehalose"
"galactose"	"xylitol" OR "xylit"
"galacturonic acid" OR "galacturonate"	"xylonic acid" OR "xylonate"
"glucaric acid" OR "saccharic acid" OR "glucarate"	"xylose"
"gluconic acid" OR "gluconate"	"xylulose"

Table S2. Concentrations in the literature for various sugars in urine.

Sugar compound	Method	Number of: Analyzed sugars	Number of: Samples	Concentration in µmol/L[a]	Normalized concentration in: µmol/mmol creatinine[a]	µmol/24 h[a]	µmol/L/mOsm/kg[a]	Literature
1,5-AG	LC	1	10	9±6[c]				[1]
	LC-MS	1	6		[2.07[d]-18.95[e]]			[2]
2,4-DHB	GC-MS	10	9		1.0 (0.3[d]-1.8[e])			[3]
Allose	GC	31	24		3.7±1.8 [1.3[d]-8[e]]			[4]
Arabinose	GC	1	13	(400) IQR: 266[d]-533[e]				[5]
	GC	31	24		16.4±8.3 [8.2-36.7[e]]			[4]
	NMR	21	28		13.3 [1.3-22.0]			[3]
	GC-MS	10	22		8.8 [0.8[d]-19.4]			[3]
	GC	1	13			[243[d]-2430[e]]		[5]
Arabitol	GC-FID	31	24		23.1±8.3 [10.2[d]-43.9]			[4]
	Others	7	12		27.3±10			[6]
	NMR	21	28		31.8 [10.2-64.3[e]]			[3]
	Others	11	9			[122[d]-485[e]]		[7]
	GC	10	6			316.8±139.2		[8]
chiro-Inositol	GC-MS	4	32		[1.6[d]-66.3[e]]	[2.4[d]-125.2[e]]		[9]
	LC	2	93			[36.1-96.0]		[10]
	GC-MS	2	10			96±17.6		[11]
Erythritol	GC-FID	18	24		26.1±16.9 [19.2-75.5[e]]			[4]
	NMR	21	28		33.4 [6.8[d]-64]			[3]
	GC	10	6			501.6±199.2		[8]
	Others	11	9			[455[d]-1060[e]]		[7]
Fructose	Others	4	1	[61.1[d]-65.5[e]]				[12]
	GC	31	24		23.9±14.8 [4.6[d]-57[e]]			[4]
	Enzymatic	2	519			[2.78[d]-1266.7[e]]		[13]
	Enzymatic/ GC	2	154			[276.53-317.44]		[14]
	LC-MS	3	20			[n.d.-2189.1]		[15]
	LC-MS	2	288			204.8±187.6 (162.1) IQR: 88.8-255.3		[16]
	Others	2	494			[55.5-88.3]		[17]
	Enzymatic	2	11			[117.7-343]		[18]
Fucose	GC	31	24		5.6[d]±3.2 [n.d.-12.3]			[4]
	n.a.	1	22		16.2±3.3			[19]
	Enzymatic	1	97		16.6±3.8			[20]
	NMR	21	28		11.8 [6.7-26.0[e]]			[3]
Galactitol	GC-MS	2	7		1.42±0.26			[21]
	GC	31	24		3.2±1.2 [1.5-5.6]			[4]
	GC-MS	2	20		1.6±0.4 (1.7) [0.6[d]-2.1]			[22]
	Others	7	12		20.1[e]±7.2			[6]
Galactose	GC-MS	2	7		0.47[d]±0.13			[21]
	GC	31	24		5.2±4.5 [n.d.-21.7]			[4]
	NMR	21	28		11.9 [5.4-25.2[e]]			[3]
	GC-MS	10	22		3.3 [1.3-8.5]			[3]
Glucaric acid	Enzymatic	1	573	[11[d]-155[e]]	[1.13-8.15]			[23]
	LC	1	38		(3.4) [<0.8-26]			[24]
	Enzymatic	1	42		[6.1-8.3]			[25]
	Enzymatic	1	20		3.2 CI: 3-3.4			[26]
	GC	4	1		0.0139[d]			[27]
	Enzymatic	1	74		(5.5) [1.7-12.4[e]]			[28]
	Enzymatic	1	30		(1.84) [0.98-4.1]			[29]
	NMR	21	28		8.4 [5.8-12.3]			[3]
	Enzymatic	1	48			26.7±10.9		[30]
	Enzymatic	1	22			26.4		[31]
	Enzymatic	1	5			[36.8-48.2[e]]		[32]

Table S2 continued.

Sugar compound	Method	Number of: Analyzed sugars	Number of: Samples	Concentration in µmol/L^{a}	Normalized concentration in: µmol/mmol creatinine^{a}	µmol/24 h^{a}	µmol/L/mOsm/kg^{a}	Literature
Glucaric acid	Enzymatic	1	15			18.4^{d}±11.5		[33]
	Enzymatic	1	6			40±9		[34]
Gluconic acid	NMR	21	28		21.5 [8.1^{d}-38.8^{e}]			[3]
	GC-MS	10	22		13.3 [8.2-26.4]			[3]
Glucose	Others	4	2	[682-5206^{e}]				[12]
	NMR	1	2	[376.1^{d}-529.6]				[35]
	NMR	21	28		37.5 [12.5-58.4^{e}]			[3]
	GC-MS	10	22		35.6 [10.3-56.7]			[3]
	GC	31	24		18.7±5.2 [3.7^{d}-27.7]			[4]
	NMR	1	5492		[19.1-28.6]			[36]
	n.a.	1	26			43.2^{d}±16.8		[37]
	n.a.	1	14			(583) IQR: 361-1409^{e}		[38]
Glucuronic acid	GC	4	1		0.4261^{d}			[27]
	NMR	21	28		9.7 [3.7-20.6^{e}]			[3]
	Others	1	60			[2225^{d}-2291^{e}]		[39]
Lactose	Enzymatic	1	52	(70.11) [43.8^{d}-409.0^{e}]				[40]
	GC	31	24		3.7±1.9 [1.3-7.4]			[4]
	NMR	21	28		11.8 [1.0^{d}-24.2^{e}]			[3]
Levoglucosan	LC-MS	1	2	[32.1-35.8]				[41]
	GC-MS	1	24	[4.3^{d}-820^{e}]	[1.0^{d}-85.8^{e}]			[42]
	NMR	21	28		10 [2.4-29]			[3]
Maltose	LC-MS	6	30	[87.6^{d}-292^{e}]				[43]
	NMR	21	28		6.0 [1.3^{d}-21.4^{e}]			[3]
Mannitol	LC-MS	6	30	[164.7^{d}-2744.7^{e}]				[43]
	GC-MS	2	3	[301.9-711.0]				[44]
	GC	31	24		18.1±13.4 [3.1^{d}-60.5]			[4]
	Others	7	12		28.8±11.8			[6]
	NMR	21	28		35.4 [5.2-85.1^{e}]			[3]
	GC	10	6			139.2±96		[8]
	Others	11	9			[122^{d}-1390^{e}]		[7]
Mannoheptulose^{f}	LC-MS	5	52		0.7^{d} [n.d.-3^{e}]			[45]
myo-Inositol	Enzymatic	1	13	76±63				[46]
	LC-MS	1	4	[39.4^{d}-84.9^{e}]				[47]
	GC	18	24		5.5±4.1 [2.3^{d}-22.7]			[4]
	Enzymatic	1	33		11.7±7.0			[48]
	NMR	21	28		22.4 [7.9-36.1]			[3]
	GC-MS	10	22		12.6 [5.1-15.3]			[3]
	GC-MS	4	32		[25.4-125.2^{e}]	[39.2^{d}-942.2^{e}]		[9]
	LC	2	93			[91-193.3]		[10]
	GC-MS	2	10			192±54		[11]
	GC-MS	3	20			183±36.1		[49]
	GC	10	6			362.4±156		[8]
Ribitol	GC	31	24		3.8±1 [1.8^{d}-5.4^{e}]			[4]
	Others	7	12		4.6±1.8			[6]
	GC	10	6			43.2^{c}±16.8		[8]
Ribose	LC		1725	(38.1) [1.53^{d}-20.89^{e}]				[50]
scyllo-Inositol	LC-MS	4	4		[5-12.5^{e}]			[51]
	NMR	21	28		4.2 [2.2-8.1]			[3]
	GC-MS	10	22		3.6 [1.9^{d}-6.9]			[3]
Sedoheptulose^{f}	LC-MS	5	52		5.7^{d} [n.d.-9^{e}]			[45]
	LC-MS	2	50		<9			[52]
Sorbitol	GC-MS	2	6	[43.9^{d}-93.3^{e}]				[44]
	GC	31	24		6.5±4.8 [2.3-26.3^{e}]			[4]
	Others	7	12		5.4±2.6			[6]
	NMR	21	28		9.9 [2.5-18.7]			[3]
	GC-MS	10	22		3.9 [1.9^{d}-5.1]			[3]
	GC	10	6			96^{d}±26.4		[8]
	GC-MS	3	20			104.8^{e}±40.6		[49]

Table S2 continued.

Sugar compound	Method	Number of: Analyzed sugars	Number of: Samples	Concentration in μmol/L[a]	Normalized concentration in: μmol/mmol creatinine[a]	μmol/ 24 h[a]	μmol/L/ mOsm/kg[a]	Literature
Sucrose	LC-MS	6	30	[204[d]-2921[e]]				[43]
	Others	4	1	[485-510.4]				[12]
	GC	31	24		8.6±6.7 [0.8[d]-30.6[e]]			[4]
	NMR	21	28		7.4 [1.4-19.5]			[3]
	GC	2	124			[49.34-78.29]		[14]
	LC-MS	3	20			[1.2[d]-716.6[e]]		[15]
	LC-MS	2	288			94.7±99.0 (71.6) IQR: 41.5-110.4		[16]
	Enzymatic	2	519			[9.6-223.9]		[13]
	Others	2	494			[55.8-93.5]		[17]
	Enzymatic	2	11			66.9-106.6		[18]
Threitol	GC	31	24		13.8±7.5 [4.8[d]-34.3[e]]			[4]
	NMR	21	28		19.3 [5.3-32.7]			[3]
	Others	11	9			[78-495[e]]		[7]
	GC	10	6			69.6[d]±26.4		[8]
Threonic acid	NMR	21	28		20.8 [10.2[d]-39.3[e]]			[3]
Xylitol	GC	31	24		4.6±3.7 [1.3-20[e]]			[4]
	Others	7	12		7.3±2.6			[6]
	GC	4	1		8.4			[27]
	NMR	21	28		8.4 [6.0-13.8]			[3]
	GC-MS	10	22		2.3 [0.9[d]-8.2]			[3]
	GC	10	6			43.2[c]±16.8		[8]
Xylose	GC	31	24		19.8±15.1 [3.7-53.6]			[4]
	NMR	21	28		20 [3.0[d]-102[e]]			[3]
	NMR	1	546				[0.596-0.637]	[53]
Xylulose	GC-MS	10	22		19.7 [6.4[d]-32.6[e]]			[3]

[a] Mean ± standard deviation; (Median); [Range]; IQR: interquartile range 25th-75th percentile; CI: 95% confidence interval; CV: coefficient of variation.
[c] Only one value; range for healthy adults for Figure 4 calculated as follows: (mean - standard deviation) – (mean + standard deviation).
[d] Used as lower limit value for healthy adults for Figure 4.
[e] Used as upper limit value for healthy adults for Figure 4.
[f] Data refer to individuals aged 0-75 years, as no separate values for adults were reported in the literature.
GC-MS: Gas chromatography coupled with (tandem/time-of-flight) mass spectrometry. LC-MS: Liquid chromatography coupled with (tandem/time-of-flight) mass spectrometry. GC: Gas (liquid) chromatography coupled with flame ionization detector. LC: Liquid chromatography coupled with UV-/refractive index/electrochemical detector. Others: Paper chromatography/ion exchange chromatography/capillary electrophoresis/ single reaction interface flow analysis/ calorimetrically/ paper-based point of care method. 1,5-AG: 1,5-Anhydroglucitol. 2,4-DHB: 2,4-Dihydroxybutyric acid. n.a.: Not available. n.d.: Not detectable.

Table S3. Concentrations in the literature for various sugars in blood, serum or plasma

Sugar compound	Method[a]	Number of: Analyzed sugars	Number of: Samples	Concentration μmol/L[b]	Literature
1,5-AG	GC-MS	5	1	119.03	[54]
	Enzymatic	1	110	(143) [70-209]	[55]
	GC-MS	8	12	94.1±29.64	[56]
	GC-MS	9	14	186.4±69.9	[57]
	Enzymatic	1	47	145.6 (149.2) Q.D:29.8	[58]
	GC	1	229	145.0±43.9	[59]
	Enzymatic	1	116	135.2±43.9	[60]
	Enzymatic	1	40	155±47 [67-285[e]]	[1]
	Enzymatic	1	3	[33.5[d]-85.3]	[61]
	Enzymatic	1	100	145.0±37.2	[62]
	Enzymatic	1	1799	112.1±31.1	[63]
	Enzymatic	1	291	(137.7) [113.9-168.1]	[64]
	Enzymatic	1	224	122.4±39.0	[65]
	Enzymatic	1	1295	106.0±39.6	[66]
	Enzymatic	1	696	188.2±55.4	[67]
	Enzymatic	1	242	(155) IQR: 128-183	[68]
	LC	8	1	85.3	[69]
	Enzymatic	1	23	138.9±40.6	[37]
Arabinose	Others	6	6	2.7[c]±0.6	[70]
Arabitol	GC-MS	8	12	2.62±0.36	[56]
	GC-MS	9	14	<2.63	[57]
	Enzymatic	1	10	[1.1-1.58]	[71]
	Enzymatic	1	12	0.8±0.6	[72]
	GC	2	24	[0.72[d]-1.3]	[73]
	LC-MS	16	20	1.18±0.11945	[74]
	GC-MS	1	39	3.42±2.23	[75]
	GC-MS	8	33	2.43±0.79	[76]
	GC	1	27	[1.05-1.45]	[77]
	n.a.	1	10	3.62[e]±0.33	[78]
chiro-Inositol	GC-MS	2	50	(0.14) IQR: 0.075[d]-0.35[e]	[79]
Erythritol	LC-MS	16	20	4.18[e]±9.17876	[74]
	GC-MS	9	14	4.1±1.6	[57]
	GC-MS	8	33	3.68[d]±1.23	[76]
Fructose	GC-MS	5	1	40.74	[54]
	Others/LC	4	1	[200.9-209.8[e]]	[12]
	LC-MS	1	40	5.4±4.5	[80]
	GC-MS	9	14	101.0±92.1	[57]
	Enzymatic	1	107	4.4±0.38	[81]
	LC-MS	16	20	6.1±3.29974	[74]
	LC-MS	7	3	[2.71[d]-62.22]	[82]
	Enzymatic	1	11	[16.65-38.85]	[83]
	GC-MS	1	23	8.1±1	[37]
Fucose	LC-MS	16	20	3.225[c]±0.78327	[74]
Galactitol	GC-MS	2	7	0.14[d]±0.03	[21]
	GC-MS	8	12	0.36±0.13	[56]
	GC-MS	8	33	0.82[e]±0.6	[76]
Galactose	GC-MS	2	7	0.2±0.03	[21]
	GC-MS	1	16	0.12[d]±0.03	[84]
	GC-MS	2	14	(200)[e]	[85]
	LC	8	1	27.75	[69]
Glucaric acid	Enzymatic	1	100	43.78[c]±9.5	[86]
Glucosamine	LC	1	12	[0.20[d]-1.2]	[87]
	LC-MS	1	82	0.359±0.263 [LOQ-1.14]	[88]
	Others	2	50	0.32	[89]
	LC	1	14	0.8628[e]±0.364	[90]
Glucose	GC-MS	5	1	4942	[54]
	LC	8	1	4996	[69]

Table S3 continued.

Sugar compound	Method[a]	Number of: Analyzed sugars	Number of: Samples	Concentration µmol/L[b]	Literature
Glucose	Others	6	6	4857±511	[91]
	LC-MS	16	20	2660±370	[74]
	LC-MS	7	3	[3024-8403]	[82]
	GC-MS	9	14	6078±1449	[57]
	LC	2	200	4550±460	[92]
	Enzymatic	1	116	[5273-6883]	[60]
	Enzymatic	1	100	[5051-5717]	[62]
	Enzymatic	1	1295	5884 CI: 5834-5995	[66]
	Enzymatic	1	449	[5200-6200]	[67]
	Enzymatic	1	23	5060±310	[37]
	Others	1	107	5200±160	[81]
	Enzymatic	1	15	[3774-5218]	[83]
	Enzymatic	1	9	5700±100	[93]
	n.a.	1	14	(5140) IQR: 4960-5290	[38]
	Enzymatic	1	17	[5560-5640]	[9]
Lactose	Others	6	6	0.61[d]±0.18	[70]
	n.a.	1	9	1.5±0.1 [f]	[94]
	GC-MS	2	14	(20)[e]	[85]
Maltose	LC-MS	16	20	1.340[c]±0.64535	[74]
Mannitol	GC-MS	8	12	1.62[d]±0.49	[56]
	LC-MS	16	20	1.675±0.184864	[74]
	GC-MS	8	33	2.25±2.47	[76]
	GC	5	6	34[e]±18	[8]
	LC	8	1	<0.55	[69]
Mannose	GC-MS	5	1	160.13[e]	[54]
	LC-MS	1	20	55.12±18.71	[95]
	GC-MS	9	14	83.8±38.85	[57]
	LC-MS	1	574	(66.1)	[96]
	Enzymatic	1	23	53.8±9.3	[97]
	Enzymatic	1	11	54.1±11.9 [36-81]	[98]
	Enzymatic	1	6	18.5±5.5	[99]
	LC	2	200	44.51±4.42	[92]
	LC-MS	1	323	34.86±20.04	[100]
	LC-MS	7	3	[3.61[d]-9.88]	[82]
	LC	8	1	<55	[69]
myo-Inositol	GC-MS	5	1	44.68	[54]
	GC-MS	8	12	20.56±5.38	[56]
	LC-MS	1	105	CI: 16.6-44.2	[101]
	GC-MS	9	14	36.08±15.0	[57]
	LC-MS	1	94	(34.5) IQR: 30-43.5	[102]
	GC-MS	2	50	(20.65) IQR: 15.76-22.86	[81]
	Enzymatic	1	20	61±12.4	[46]
	GC	4	5	19.9±2.5[f]	[103]
	n.a.	1	n.a.	24.5	[104]
	LC	8	1	2.5[d]	[69]
	GC	5	6	63[e]±24	[8]
Ribitol	GC-MS	8	12	0.46[e]±0.085	[56]
	GC-MS	9	14	<2.62	[57]
	GC-MS	8	33	0.39[d]±0.13	[76]
Ribose	Others	6	6	2.0[d]±0.33	[70]
	LC-MS	16	20	10.05±3.96025	[74]
	Others	1	8	70.61 [0-233[e]]	[105]
	LC-MS	7	3	[18.85-22.11]	[82]
scyllo-Inositol	GC	4	5	0.46[c]±0.13[f]	[103]
Sedoheptulose[g]	LC-MS	1	35	0.4[c]±0.2	[106]
Sorbitol	LC-MS	9	14	<2.2	[57]
	GC-MS	8	12	1.1±0.37	[56]
	Enzymatic	1	9	4.1±0.4	[93]

Table S3 continued.

Sugar compound	Method[a]	Number of: Analyzed sugars	Number of: Samples	Concentration *μ*mol/L[b]	Literature
Sorbitol	LC-MS	2	16	0.4[d]±0.2	[107]
	Enzymatic	1	4	12.6±2	[108]
	LC-MS	1	20	2.5±0.5	[109]
	GC-MS	8	33	0.88±0.60	[76]
	GC	5	6	18[e]±7	[8]
	LC	8	1	<4.4	[69]
Sucrose	LC-MS	16	20	0.14694±0.2089[h]	[74]
Threitol	GC-MS	8	33	1.63[c]±0.49	[76]
Xylitol	GC-MS	8	12	0.677[e]±0.11	[56]
	GC-MS	8	33	0.33[d]±0.13	[76]
Xylose	Others	6	6	1.0[d]±0.2	[70]
	LC-MS	16	20	3.31[e]±0.65	[74]

[a] Mean ± standard deviation; (Median); [Range]; IQR: interquartile range 25th-75th percentile; CI: 95% confidence interval; CV: coefficient of variation; Q.D.: quartile deviation.

[c] Only one value; range for healthy adults for Figure 4 calculated as follows: (mean - standard deviation) – (mean + standard deviation).

[d] Used as lower limit value for healthy adults for Figure 4.

[e] Used as upper limit value for healthy adults for Figure 4.

[f] Mean ± standard error.

[g] Data refer to individuals aged 0-17 years, as no separate values for adults were reported in the literature.

[h] Value not shown in Figure 4 due to the standard deviation is higher than the mean.

GC-MS: Gas chromatography coupled with (tandem/time-of-flight) mass spectrometry. LC-MS: Liquid chromatography coupled with (tandem/time-of-flight) mass spectrometry. GC: Gas (liquid) chromatography coupled with flame ionization detector. LC: Liquid chromatography coupled with UV-/refractive index/electrochemical detector. Others: Paper chromatography/ion exchange chromatography/capillary electrophoresis/ single reaction interface flow analysis/ calorimetrically/ paper-based point of care method. 1,5-AG: 1,5-Anhydroglucitol. n.a.: Not available. n.d.: Not detectable.

S2 Chemicals and materials

Table S4. List of the applied chemicals and reference standards including information on purity and supplier.

Substance	Purity	Substance	Purity	Substance	Purity
**Omicron Biochemicals, USA**		**Carl Roth, Germany**		**Sigma-Aldrich, USA**	
1,5-Anhydroglucitol $^{13}C_6$	99.6%	Fucose	>95%	2,4-Dihydroxybutyric acid	>95%
Arabinose $^{13}C_5$	100%	Galactitol	>99%	Arabinose	>98%
Arabitol $^{13}C_5$	100%	Galactosamine	>97%	Erythronic acid	n.a.
Erythritol $^{13}C_4$	99.7%	Glucuronic acid	>98%	Erythronolactone	95%
Erythrose $^{13}C_4$	n.a.	Mannose	>98.5%	Fructose	99%
Fucose $^{13}C_6$	99.3%	*N*-Acetylgalactosamine	>99%	Galactose	>99%
Galactitol $^{13}C_6$	99.1%	*N*-Acetylglucosamine	>99%	Glucaric acid	>98,5%
Galactose $^{13}C_6$	100%	Ribitol	>98%	Gluconic acid	>99%
Gluconic acid $^{13}C_6$	100%	Ribose	>98%	Glucosamine	>99%
Glucosamine $^{13}C_6$	n.a.	Xylose	>99%	Glucose	>99.5%
Glucuronic acid $^{13}C_6$	98.6%	*N*-Methyl-*N*-trimethylsilyltrifluoracetamide	>99%	Lactose	99.5%
Lactose $^{13}C_{12}$	99.2%			Lactose	99.5%
Levoglucosan $^{13}C_6$	99.5%	**Carbosynth/Biosynth, UK**		Maltose	99%
Maltose $^{13}C_{12}$	99.7%	1,5-Anhydroglucitol	97%	Mannitol	a.c.
Mannitol $^{13}C_6$	99.7%	2-Deoxyribonic acid-1,4-lactone	98%	*myo*-Inositol	99%
Mannoheptulose $^{13}C_7$	99.6%	5-Deoxyarabonic acid-1,4-lactone	97%	*N*-Acetylmannosamine	>98%
Mannose $^{13}C_6$	99.9%	Allose	99.7%	Ribonic acid	n.a.
N-Acetylglucosamine $^{13}C_6$	NA	Arabino-1,4-lactone	99.8%	Sedoheptulose	>95%
Psicose $^{13}C_6$	99.0%	*chiro*-Inositol	98%	Sucrose	>99.5%
Ribitol $^{13}C_5$	99.7%	Erythrose	95%	Threitol	99%
Ribose $^{13}C_5$	99.9%	Ethyl glucoside	98%	Threonic acid	97%
Sorbitol $^{13}C_6$	99.9%	Fucitol	95%	Trehalose	>99%
Tagatose $^{13}C_6$	99.9%	Mannoheptulose	98%	Xylitol	>99%
Trehalose $^{13}C_{12}$	100%	Mannonic acid-1,4-lactone	99.2%	Methyl decanoate	99%
Xylitol $^{13}C_5$	100%	*scyllo*-Inositol	98%	Methyl tetradecanoate	>99%
Xylose $^{13}C_5$	99.5%	Xylonic acid	97%	Methyl eicosanoate	>99%
Xylulose $^{13}C_5$	99.9%	Xylulose	n.a.	Methyl tetracosanoate	≥99%
**Fluka, Swiss**		Saccharose $^{13}C_{12}$	98.0%	Dodecan	>99%
Heptadecan	n.a.	**Merck, Germany**		Tridecan	>99%
Octodecan	n.a.	Galacturonic acid	Bc	Tetradecan	>99%
Nonadecan	n.a.	Rhamnose	>99%	Docosan	n.a.
Eicosan	n.a.	Sorbitol	99%	Tricosan	n.a.
Heneicosan	n.a.	Methyl octanoate	GC	Nonacosan	n.a.
Tetracosan	n.a.	Methyl dodecanoate	0.99	**ABCR, Germany**	
Pentacosan	n.a.	Methyl octadecanoate	> 99%	Arabinonic acid	99%
Hexacosan	n.a.	Acetone	≥99.8%	Erythritol	99%
Heptacosan	n.a.	Heptane	≥99.3%	Levoglucosan	99%
Octacosan	n.a.	Methanol	>99.8%	Psicose	98%
Triacontan	n.a.	Octan	0.995	Ribono-1,4-lactone	95%
Methyl heptanoate	>99.8%	Nonan	0.995	Tagatose	99%
Methyl hexadecanoate	>99%	Decan	0.995	**Campro Scientific, Germany**	
Methyl docosanoate	>99%	Undecan	n.a.	Fructose $^{13}C_6$	n.a.
Methyl hexacosanoate	95%	Pentadecan	n.a.	Glucose $^{13}C_6$	n.a.
Methyl octacosanoate	>99%	Hexadecan	n.a.	**Thermo Fisher Scientific, USA**	
**Tokio Chemical Industry, Japan**		**CDN Isotopes, Canada**		Pyridine, anhydrous	>99.5%
Arabitol	>98%	*myo*-Inositol D_6	98.3%		

n.a. = Not available. Bc = Biochemical grade. GC = Gas chromatography grade. a.c. = Analytical grade.

Table S5. List of used equipment including information on supplier and application.

**Instrument**	**Supplier**	**Applied for**
Model 2020	Advanced Instruments, Inc, USA	Determination of osmolality
Compact Star CSC	VWR, USA	Urine centrifugation
Centrifuge 5403	Eppendorf, Germany	Blood centrifugation
Vortex Genie 2	Scientific industries, USA	Homogenization
SpeedVac RVC 2-25 CDplus	Christ, Germany	Evaporation
Thermomixer comfort	Eppendorf, Germany	Derivatization
Labostar	Siemens, Germany	Ultra pure water

S3 Calibration and internal standards

Table S6. Concentration of each sugar in the different calibration levels and spiking solutions [μmol/L] for the validation measurement series.

	Urine quantification											Serum quantification									
	Calibration levels [μmol/L]								Spiking solutions for accuracy [μmol/L]			Calibration levels [μmol/L]							Spiking solutions for accuracy [μmol/L]		
**Sugar compound**	**1**	**2**	**3**	**4**	**5**	**6**	**7**	**8**	**1**	**2**	**3**	**1**	**2**	**3**	**4**	**5**	**6**	**7**	**1**	**2**	**3**
1,5-Anhydroglucitol	0.03	0.1	0.5	1	2	3	4	5	0.5	3	8	300	250	200	150	100	50	30	150	100	75
2,4-Dihydroxybutyric acid	38.1	28.6	19.1	14.3	9.5	4.8	2	0.5	57.2	23.8	2.9	0.1	0.71	1.4	2.4	4.8	7.1	9.5	0.71	2.4	7.1
2-Deoxyribonic acid-1,4-lactone	0.05	0.5	2.5	5	10	20	30	50	1.4	15	60	--	--	--	--	--	--	--	--	--	--
5-Deoxyarabonic acid-1,4-lactone	0.05	0.5	2.5	5	10	20	30	50	1.4	15	60	--	--	--	--	--	--	--	--	--	--
Allose	--	--	--	--	--	--	--	--	--	--	--	0.02	0.1	0.5	1	2.5	5	7.5	0.5	1	4
Arabino-1,4-lactone	1	7.5	15	25	50	75	100	125	8	60	200	--	--	--	--	--	--	--	--	--	--
Arabinonic acid	--	--	--	--	--	--	--	--				0.04	0.15	0.25	1	2	4	10	0.2	1	4
Arabinose	1	7.5	15	25	50	75	100	125	8	60	200	0.02	0.1	0.5	1	2.5	5	7.5	0.5	1	4
Arabitol	1	7.5	15	25	50	75	100	125	8	60	200	0.1	0.75	1.5	2.5	5	7.5	10	0.75	2.5	7.5
chiro-Inositol	0.01	0.1	1	2.5	5	7.5	10	15	0.6	6	20	--	--	--	--	--	--	--	--	--	--
Erythritol	200	150	100	75	50	25	10	1	300	120	16	0.1	0.75	1.5	2.5	5	7.5	10	0.75	2.5	7.5
Erythronic acid	--	--	--	--	--	--	--	--	--	--	--	2	5	7.5	10	15	20	30	5	10	20
Erythronolactone	1	7.5	15	25	50	75	100	125	8	60	200	--	--	--	--	--	--	--	--	--	--
Erythrose	0.03	0.12	0.6	1.2	2.4	3.6	4.8	6	0.6	3.6	9.6	--	--	--	--	--	--	--	--	--	--
Ethyl glucoside	--	--	--	--	--	--	--	--	--	--	--	0.02	0.1	0.5	1	2.5	5	7.5	0.5	1	4
Fructose	0.75	5.6	11.3	18.8	37.6	56.4	75.2	94	6	45.1	150.4	0.1	2.5	10	25	50	100	200	5	15	100
Fucitol	0.03	0.1	0.5	1	2	3	4	5	0.5	3	8	--	--	--	--	--	--	--	--	--	--
Fucose	40	30	20	15	10	5	2	0.5	60	25	3	0.02	0.075	0.125	0.5	1	2	5	0.1	0.5	2
Galactitol	0.05	0.5	2.5	5	10	20	30	50	1.4	15	60	0.02	0.075	0.125	0.5	1	2	5	0.1	0.5	2
Galactosamine	0.03	0.1	0.5	1	2	3	4	5	0.5	3	8	--	--	--	--	--	--	--	--	--	--
Galactose	50	30	15	10	5	2.5	0.5	0.01	60	15	0.6	0.02	0.1	0.5	1	2.5	5	7.5	0.5	1	4
Galacturonic acid	0.03	0.1	0.5	1	2	3	4	5	0.5	3	8	--	--	--	--	--	--	--	--	--	--
Glucaric acid	40	30	20	15	10	5	2	0.5	60	25	3	0.02	0.075	0.125	0.5	1	2	5	0.1	0.5	2
Gluconic acid	100	75	50	25	10	5	0.5	0.05	150	30	0.8	0.1	0.75	1.5	2.5	5	7.5	10	0.75	2.5	7.5
Glucosamine	0.03	0.1	0.5	1	2	3	4	5	0.5	3	8	0.02	0.075	0.125	0.5	1	2	5	0.1	0.5	2
Glucose[a]	75	50	25	15	10	5	2	0.5	100	25	2.5	2000	4000	6000	8000	10000	--	--	--	--	--
Glucuronic acid	40	30	20	15	10	5	2	0.5	60	25	3	0.1	0.75	1.5	2.5	5	7.5	10	0.75	2.5	7.5

Table S6 continued.

	Urine quantification											Serum quantification									
	Calibration levels [µmol/L]								Spiking solutions for accuracy [µmol/L]			Calibration levels [µmol/L]							Spiking solutions for accuracy [µmol/L]		
**Sugar compound**	**1**	**2**	**3**	**4**	**5**	**6**	**7**	**8**	**1**	**2**	**3**	**1**	**2**	**3**	**4**	**5**	**6**	**7**	**1**	**2**	**3**
Lactose	0.05	0.5	2.5	5	10	20	30	50	1.4	15	60	0.009	0.035	0.06	0.24	0.47	0.94	2.4	0.1	0.5	2
Levoglucosan	50	30	15	10	5	2.5	0.5	0.01	60	15	0.6	0.02	0.075	0.125	0.5	1	2	5	0.1	0.5	2
Maltose	0.01	0.1	1	2.5	5	7.5	10	15	0.6	6	20	0.01	0.03	0.06	0.23	0.46	0.9	2.3	0.05	0.24	1
Mannitol	200	150	100	75	50	25	10	1	300	120	16	0.02	0.1	0.5	1	2.5	5	7.5	0.5	1	4
Mannoheptulose	0.05	0.5	2.5	5	10	20	30	50	1.4	15	60	0.02	0.04	0.06	0.08	0.1	0.2	0.3	0.04	0.08	0.2
Mannonic acid-1,4-lactone	1	7.5	15	25	50	75	100	125	8	60	200	--	--	--	--	--	--	--	--	--	--
Mannose	25	20	15	10	5	2.5	1	0.1	40	6	1	75	50	25	15	5	0.5	0.05	40	15	2
myo-Inositol	40	30	20	15	10	5	2	0.5	60	25	3	75	50	25	15	5	0.5	0.05	40	15	2
N-Acetylgalactosamine	0.05	0.5	1	2	4	6	8	10	1	6	16	--	--	--	--	--	--	--	--	--	--
N-Acetylglucosamine	0.05	0.5	1	2	4	6	8	10	1	6	16	0.02	0.075	0.125	0.5	1	2	5	0.1	0.5	2
N-Acetylmannosamine	0.05	0.5	1	2	4	6	8	10	1	6	16	--	--	--	--	--	--	--	--	--	--
Psicose	1	7.5	15	25	50	75	100	125	8	60	200	0.014	0.053	0.088	0.35	0.71	1.41	3.5	0.1	0.5	2
Rhamnose	0.03	0.1	0.5	1	2	3	4	5	0.5	3	8	--	--	--	--	--	--	--	--	--	--
Ribitol	40	30	20	15	10	5	2	0.5	60	25	3	0.02	0.075	0.125	0.5	1	2	5	0.1	0.5	2
Ribonic acid	--	--	--	--	--	--	--	--	--	--	--	0.1	0.75	1.5	2.5	5	7.5	10	0.75	2.5	7.5
Ribono-1,4-lactone	40	30	20	15	10	5	2	0.5	60	25	3	--	--	--	--	--	--	--	--	--	--
Ribose	40	30	20	15	10	5	2	0.5	60	25	3	0.02	0.1	0.5	1	2.5	5	7.5	0.5	1	4
scyllo-Inositol	0.05	0.5	2.5	5	10	20	30	50	1.4	15	60	0.02	0.1	0.5	1	2.5	5	7.5	0.5	1	4
Sedoheptulose	0.05	0.5	2.5	5	10	20	30	50	1.4	15	60	0.02	0.075	0.125	0.5	1	2	5	0.1	0.5	2
Sorbitol	0.05	0.5	2.5	5	10	20	30	50	1.4	15	60	0.02	0.075	0.125	0.5	1	2	5	0.1	0.5	2
Sucrose	100	75	50	25	10	5	0.5	0.05	150	30	0.8	0.02	0.1	0.5	1	2.5	5	7.5	0.5	1	4
Tagatose	0.03	0.1	0.5	1	2	3	4	5	0.5	3	8	--	--	--	--	--	--	--	--	--	--
Threitol	1	7.5	15	25	50	75	100	125	8	60	200	0.1	0.75	1.5	2.5	5	7.5	10	0.54	1.8	5.4
Threonic acid	80	60	40	30	20	10	4	1	120	50	6	4	10	15	20	30	40	60	10	20	40
Trehalose	0.03	0.1	0.5	1	2	3	4	5	0.5	3	8	0.02	0.04	0.06	0.08	0.1	0.2	0.3	0.04	0.08	0.2
Xylitol	75	50	25	15	10	5	2	0.5	100	25	2.5	0.02	0.1	0.5	1	2.5	5	7.5	0.5	1	4
Xylonic acid	80	60	40	30	20	10	4	1	120	50	6	0.2	1.5	3	5	10	15	20	1.5	5	15
Xylose	200	150	100	75	50	25	10	1	300	120	16	75	50	25	15	5	0.5	0.05	40	15	2
Xylulose	25	20	15	10	5	2.5	1	0.1	40	6	1	0.02	0.075	0.125	0.5	1	2	5	0.1	0.5	2

[a] For the quantification in blood separate calibration levels for glucose were used due to the high concentration of glucose.

Table S7. Concentration of each sugar in the different calibration levels and spiking solutions [μmol/L] for the application measurement series.

	Urine quantification									Serum quantification								
	Calibration levels [μmol/L]							Spiking solutions for accuracy [μmol/L]		Calibration levels [μmol/L]							Spiking solutions for accuracy [μmol/L]	
**Sugar compound**	**1**	**2**	**3**	**4**	**5**	**6**	**7**	**1**	**2**	**1**	**2**	**3**	**4**	**5**	**6**	**7**	**1**	**2**
1,5-Anhydroglucitol	0.01	0.05	0.1	1	2	3	5	0.05	3	300	250	200	150	100	50	30	100	150
2,4-Dihydroxybutyric acid	0.05	0.5	2.4	4.8	7.1	9.5	14.3	0.5	9.5	0.1	0.7	1.4	2.4	4.8	7.1	9.5	2.4	7.1
Allose	--	--	--	--	--	--	--	--	--	0.02	0.1	0.5	1	2.5	5	7.5	1	4
Arabinonic acid	--	--	--	--	--	--	--	--	--	0.04	0.15	0.25	1	2	4	10	1	4
Arabinose	100	75	50	30	15	7.5	1	75	7.5	0.02	0.1	0.5	1	2.5	5	7.5	1	4
Arabitol	150	100	50	30	20	10	2	100	10	0.1	0.75	1.5	2.5	5	7.5	10	2.5	7.5
chiro-Inositol	0.01	0.05	0.1	1	2	3	5	0.05	3	--	--	--	--	--	--	--	--	--
Erythritol	150	100	50	30	20	10	2	100	10	0.1	0.75	1.5	2.5	5	7.5	10	2.5	7.5
Erythronic acid	--	--	--	--	--	--	--	--	--	2	5	7.5	10	15	20	30	10	20
Erythrose	0.01	0.06	0.13	1.2	2.4	3.6	6	0.06	3.6	--	--	--	--	--	--	--	--	--
Ethyl glucoside	--	--	--	--	--	--	--	--	--	--	--	--	--	--	--	--	--	--
Fructose	0.1	1	2.5	5	7.5	15	30	1	15	0.1	2.5	10	25	50	100	200	15	100
Fucitol	0.01	0.05	0.1	1	2	3	5	0.05	3	--	--	--	--	--	--	--	--	--
Fucose	0.5	2.5	5	10	20	30	40	2.5	30	0.02	0.075	0.125	0.5	1	2	5	0.5	2
Galactitol	0.05	0.5	2.5	5	7.5	10	15	0.5	10	0.02	0.075	0.125	0.5	1	2	5	0.5	2
Galactose	0.01	0.5	5	25	50	75	100	0.5	75	0.02	0.1	0.5	1	2.5	5	7.5	1	4
Galacturonic acid	0.01	0.05	0.1	1	2	3	5	0.05	3	--	--	--	--	--	--	--	--	--
Glucaric acid	0.5	2.5	5	10	20	30	40	2.5	30	--	--	--	--	--	--	--	--	--
Gluconic acid	100	75	50	30	15	7.5	1	75	7.5	0.1	0.75	1.5	2.5	5	7.5	10	2.5	7.5
Glucose[a]	1	2.5	5	10	25	50	75	2.5	50	2000	4000	6000	8000	10000	--	--	--	--
Glucuronic acid	0.5	2.5	5	10	20	30	40	2.5	30	0.1	0.75	1.5	2.5	5	7.5	10	2.5	7.5
Lactose	0.01	0.5	5	25	50	75	100	0.5	75	0.009	0.035	0.06	0.24	0.47	0.94	2.4	0.5	2
Levoglucosan	0.01	0.1	1	4	7	10	15	0.1	10	0.02	0.075	0.125	0.5	1	2	5	0.5	2
Maltose	0.01	0.1	1	4	7	10	15	0.1	10	0.01	0.03	0.06	0.23	0.46	0.9	2.3	0.24	1
Mannitol	225	175	125	75	25	12.5	1	175	12.5	0.02	0.1	0.5	1	2.5	5	7.5	1	4
Mannoheptulose	0.05	0.5	2.5	5	7.5	10	15	0.5	10	0.02	0.04	0.06	0.08	0.1	0.2	0.3	0.08	0.2

Table S7 continued.

	Urine quantification									Serum quantification								
	Calibration levels [μmol/L]							Spiking solutions for accuracy [μmol/L]		Calibration levels [μmol/L]							Spiking solutions for accuracy [μmol/L]	
**Sugar compound**	**1**	**2**	**3**	**4**	**5**	**6**	**7**	**1**	**2**	**1**	**2**	**3**	**4**	**5**	**6**	**7**	**1**	**2**
Mannose	0.05	0.5	2.5	5	7.5	10	15	0.5	10	75	50	25	15	5	0.5	0.05	15	40
myo-Inositol	0.5	2.5	5	10	20	30	40	2.5	30	75	50	25	15	5	0.5	0.05	15	40
Psicose	1	2.5	5	10	25	50	75	2.5	50	0.014	0.053	0.088	0.35	0.71	1.41	3.5	0.5	2
Rhamnose	0.01	0.05	0.1	1	2	3	5	0.05	3	--	--	--	--	--	--	--	--	--
Ribitol	0.05	0.5	2.5	5	7.5	10	15	0.5	10	0.02	0.075	0.125	0.5	1	2	5	0.5	2
Ribonic acid	--	--	--	--	--	--	--	--	--	0.1	0.75	1.5	2.5	5	7.5	10	2.5	7.5
Ribose	0.05	0.5	2.5	5	7.5	10	15	0.5	10	0.02	0.1	0.5	1	2.5	5	7.5	1	4
scyllo-Inositol	--	--	--	--	--	--	--	--	--	0.02	0.1	0.5	1	2.5	5	7.5	1	4
Sedoheptulose	0.1	1	2.5	5	7.5	15	30	1	15	0.02	0.075	0.125	0.5	1	2	5	0.5	2
Sorbitol	0.05	0.5	2.5	5	7.5	10	15	0.5	10	--	--	--	--	--	--	--	--	--
Sucrose	0.01	0.1	1	4	7	10	15	0.1	10	0.02	0.1	0.5	1	2.5	5	7.5	1	4
Tagatose	0.01	0.05	0.1	1	2	3	5	0.05	3	--	--	--	--	--	--	--	--	--
Threitol	0.66	1.65	3.3	6.6	16.5	33	49.5	2.5	50	0.1	0.75	1.5	2.5	5	7.5	10	1.8	5.4
Threonic acid	1	5	10	20	40	60	80	5	60	--	--	--	--	--	--	--	--	--
Trehalose	0.01	0.05	0.1	1	2	3	5	0.05	3	0.02	0.04	0.06	0.08	0.1	0.2	0.3	0.08	0.2
Xylitol	0.0345	0.345	1.725	3.45	5.175	6.9	10.35	0.5	10	0.02	0.1	0.5	1	2.5	5	7.5	1	4
Xylonic acid	--	--	--	--	--	--	--	--	--	0.2	1.5	3	5	10	15	20	5	15
Xylose	100	75	50	30	15	7.5	1	75	7.5	75	50	25	15	5	0.5	0.05	15	40
Xylulose	0.05	0.5	2.5	5	7.5	10	15	0.5	10	0.02	0.075	0.125	0.5	1	2	5	0.5	2

[a] For the quantification in blood separate calibration levels for glucose were used due to the high concentration of glucose.

Table S8. Concentration of the ISTD [μmol/L] for the quantification of urine and serum for the validation and application measurement series

Concentrations of ISTD [μmol/L]							
	Urine		Serum		Urine		Serum
ISTD	Validation	Application	Validation+ Application	ISTD	Validation	Application	Validation+ Application
1,5-Anhydroglucitol $^{13}C_6$	2	2	250	Mannitol $^{13}C_6$	80	70	1
Arabinose $^{13}C_5$	30	15	2	Mannoheptulose $^{13}C_7$	2	1	0.15
Arabitol $^{13}C_5$	60	30	4	Mannose $^{13}C_6$	5	4	60
Erythritol $^{13}C_4$	80	60	8	*myo*-Inositol D_6	20	10	50
Erythrose $^{13}C_4$	1	0.4		*N*-Acetylglucosamine $^{13}C_6$	2	8	1
Fructose $^{13}C_6$	10	10	40	Psicose $^{13}C_6$	40	15	1
Fucose $^{13}C_6$	16	9	1.5	Ribitol $^{13}C_5$	10	6	1
Galactitol $^{13}C_6$	10	5	1	Ribose $^{13}C_5$	10	5	1
Galactose $^{13}C_6$	5	4	1	Sucrose $^{13}C_{12}$	10	2	1
Gluconic acid $^{13}C_6$	50	50	4	Sorbitol $^{13}C_6$	10	5	1
Glucose $^{13}C_6$	50	30	8000	Tagatose $^{13}C_6$	2	1.5	--
Glucosamine $^{13}C_6$	2	2	1	Trehalose $^{13}C_{12}$	1.5	1	0.15
Glucuronic acid $^{13}C_6$	30	30	4	Xylitol $^{13}C_5$	10	6	1
Lactose $^{13}C_{12}$	7	15	1	Xylose $^{13}C_5$	50	15	2
Levoglucosan $^{13}C_6$	5	2	1	Xylulose $^{13}C_5$	10	7	1.6
Maltose $^{13}C_{12}$	2	1	1.6				

ISTD: Isotopically labeled internal standards.

S4 Osmolality of urine samples

Table S9. Osmolality of urine samples.

Sample	Osmolality [mOsm/kg]	Sample	Osmolality [mOsm/kg]	Sample	Osmolality [mOsm/kg]
QC sample	170.5	Study sample 12	287.5	Study sample 27	354.5
Accuracy 1	278	Study sample 13	820	Study sample 28	346
Accuracy 2	816	Study sample 14	240.5	Study sample 29	179
Accuracy 3	336.5	Study sample 15	288	Study sample 30	284
Study sample 1	462.5	Study sample 16	343.5	Study sample 31	374
Study sample 2	356	Study sample 17	314.5	Study sample 32	385.5
Study sample 3	640	Study sample 18	223	Study sample 33	497
Study sample 4	530	Study sample 19	458	Study sample 34	286.5
Study sample 5	395	Study sample 20	601.5	Study sample 35	118
Study sample 6	829	Study sample 21	182	Study sample 36	425.5
Study sample 7	433	Study sample 22	351	Study sample 37	307.5
Study sample 8	336.5	Study sample 23	304.5	Study sample 38	816
Study sample 9	208	Study sample 24	475.5	Study sample 39	248
Study sample 10	256	Study sample 25	688.5	Study sample 40	937.5
Study sample 11	559	Study sample 26	109		

S5 GC×GC-MS and GC×GC-qTOF analysis

Table S10. Mass (*m/z*) and time range parameters for MS of urine and serum.

	Start time [min]	Stop time [min]	Scan rate [Hz]	Start *m/z*	Stop *m/z*
**Urine**					
	7	13.3	100	210	324
	13.3	34.8	100	203	317
	34.8	65.1	100	230	344
	65.1	74.99	100	306	420
**Serum**					
	8	27.3	100	203	317
	27.3	55.2	100	230	344
	55.2	68.99	100	306	420

Table S11. GC and MS parameter for the GC×GC-qTOF analysis.

**Parameter**	
Colum combination	^{1}D: Rxi-5Sil MS (Restek, Germany; 30(+10 m × 0.25 mm ID × 0.25 µm film thickness)<br>^{2}D: BPX50 (Trajan, Australia; 2 m (thereof 0.9 m as modulator loop and 0.75 m for ^{2}D separation) × 0.15 mm ID × 0.15 µm film thickness)
Linear velocity	24.54 cm/sec
Initial head pressures	143.23 kPa
Injection mode	Hot injection at 250 °C
Injection volume	1.2µL
Split ratio	1:2
Oven temperature program	120 °C→ 3 °C/min→ 340°C; total run time: 73.33 min
Modulation time	2.5 s
Hot jet pulse time	0.35 s
Cold jet mass flow program	40% (3 min) → 5% (73 min)
Ion source temperature	340 °C
Interface temperature	300 °C
Ionization conditions	EI: 70 eV (Emission current: 10 µA)<br>Low Energy EI: 20 eV (Emission current: 1 µA)<br>Low Energy EI: 12 eV (Emission current: 1 µA)
m/z range	60 - 1200
Collision gas	Nitrogen (1 mL/min)
Quench gas	Helium (4 mL/min)
Acquisition rate	50 spectra/sec
Acquisition time	20 ms/spectrum
Scan rate	50 Hz
Data storage	Centroid
Data acquisition time	4.8 -73.3 min
Program used for evaluation	Agilent MassHunter Workstation Qualitative Analysis Version 10.0 (Agilent, USA)

S6 Data processing

Table S12. Parameters for the integration method for urine and serum using AnalyzerPro XD. The integration of the urine measurements was divided into two parts, from 7-66.2 min and 66.2-74.99 min.

	**Urine integration 1**	**Urine integration 2**	**Serum integration**
	Constraints		
Process as	Features	Features	Features
Reject masses	281, 282, 283, 341, 342, 354, 355, 356, 357, 385, 400, 401, 402, 403, 414, 415, 416, 417	320, 321, 322, 324, 325, 326, 327, 328, 329, 330, 332, 333, 334, 335, 336, 337, 338, 339, 340, 341, 342, 343, 344, 345, 346, 347, 348, 349, 350, 351, 352, 353, 354, 355, 356, 357, 358, 359, 360, 362, 363, 364, 365, 366	281, 282, 283, 341, 342, 354, 355, 356, 357, 385, 400, 401, 402, 403, 414, 415, 416, 417
Mass range (*m/z*)	203-421	319-367	203-421
Processed Retention time range (min)	7-66.2	66.2-74.99	8-68.99
	Detection		
Area threshold	300	500	300
Height threshold	1250	1250	1250
Temporal resolution	Minimum	Minimum	Minimum
Scan window	5	9	5
Signal to noise	30	30	30
Smoothing	9	9	9
Fronting (%)	0	0	0
Tailing (%)	0	0	0
Width (min)	0.0007	0.0007	0.0007

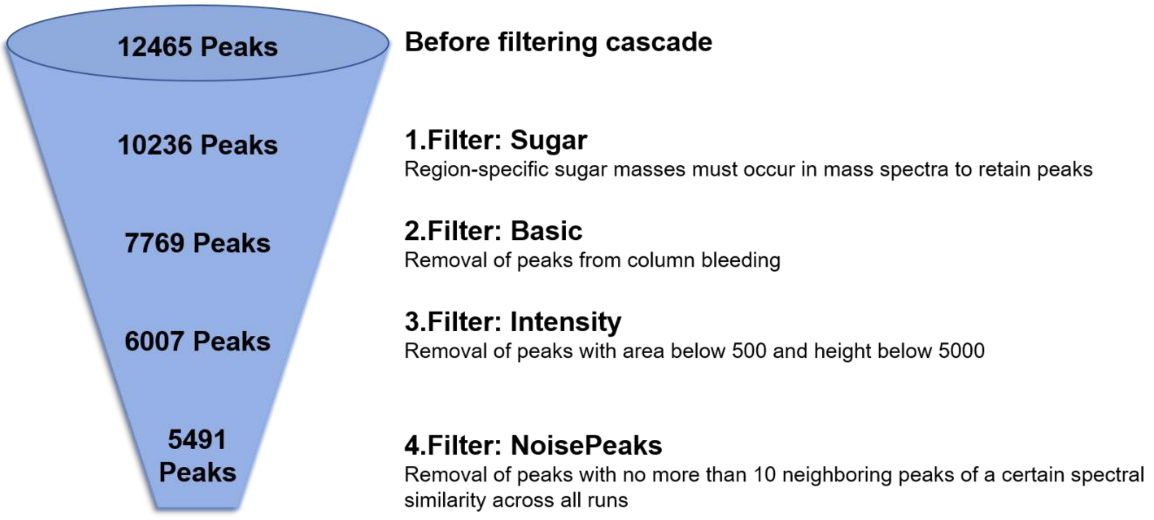

Figure S1. Exemplary data filtering cascade for one urinary QC sample.

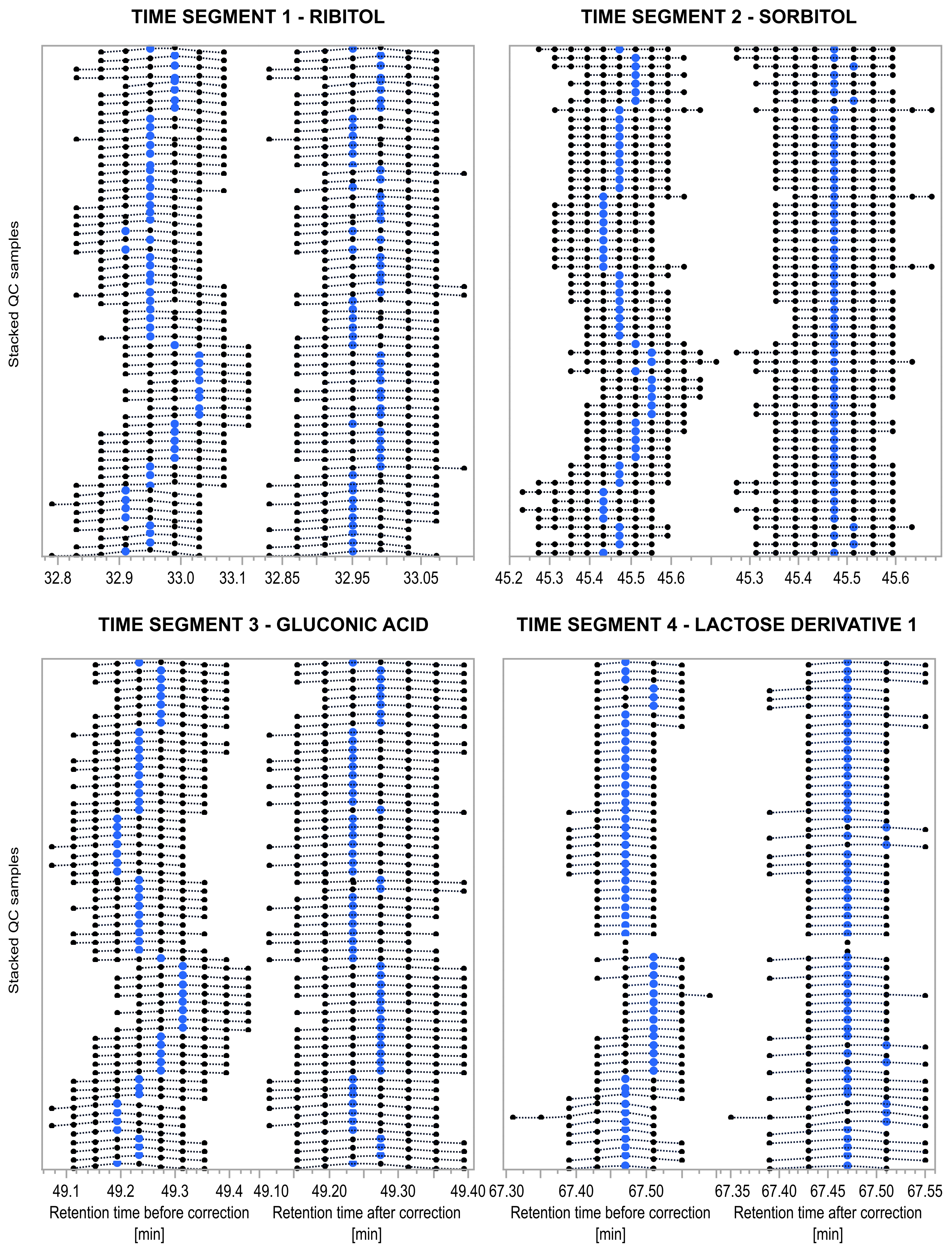

Figure S2. Comparison of the retention times before and after correction in the QC samples for four sugars in the different time segments. A point represents a modulation and the blue point represents the highest modulation.

Table S13. ISTD used for correction for RT-shift in the respective time segments.

	Urine	Serum
Time segment 1	7-37.15 min	8-32 min
ISTD	Xylose $^{13}C_5$ derivative 1, arabinose $^{13}C_5$, xylulose $^{13}C_5$, ribose $^{13}C_5$, levoglucosan $^{13}C_6$, xylitol $^{13}C_5$, ribitol $^{13}C_5$, fucose $^{13}C_6$ derivative 1 and 2	Erythritol $^{13}C_4$, xylose $^{13}C_5$ derivative 1 and 2, arabinose $^{13}C_5$, xylulose $^{13}C_5$, ribose $^{13}C_5$, levoglucosan $^{13}C_6$, xylitol $^{13}C_5$, arabitol $^{13}C_5$, ribitol $^{13}C_5$, fucose $^{13}C_6$ derivative 1
Time segment 2	37.15-48.45 min	32-40.9 min
ISTD	Fructose $^{13}C_6$ derivative 2, delta-gluconolactone $^{13}C_6$, mannose $^{13}C_6$ derivative 1, galactose $^{13}C_6$ derivative 1, glucose $^{13}C_6$ derivative 2, glucuronic acid $^{13}C_6$ derivative 1 and 2, sorbitol $^{13}C_6$, galactitol $^{13}C_6$	1,5-Anhydroglucitol $^{13}C_6$, psicose $^{13}C_6$ derivative 1, fructose $^{13}C_6$ derivative 2, delta-gluconolactone $^{13}C_6$, mannose $^{13}C_6$ derivative 1, galactose $^{13}C_6$ derivative 1, mannitol $^{13}C_6$, glucuronic acid $^{13}C_6$ derivative 1, sorbitol $^{13}C_6$, galactitol $^{13}C_6$
Time segment 3	48.45-64 min	40.9-55.2 min
ISTD	Gluconic acid $^{13}C_6$, *myo*-inositol D_6, mannoheptulose $^{13}C_7$	Gluconic acid $^{13}C_6$, *N*-acetylglucosamine $^{13}C_6$ derivative 1, *myo*-inositol D_6
Time segment 4	64-74.99 min	55.2-68.99 min
ISTD	Lactose $^{13}C_{12}$ derivative 1 and 2	Sucrose $^{13}C_{12}$, lactose $^{13}C_{12}$ derivative 1, maltose $^{13}C_{12}$ derivative 1

ISTD: Isotopically labeled internal standards.

Table S14. Quantified sugar compounds in urine, their corresponding RI, as well as selected quantification ion, selected ISTD, and their corresponding quantification ion for the ISTD.

**Sugar compound**[a]	**RI – alkane**[b]	**RI – FAME**[c]	**Quantification ion sugar (*m/z*)**	**Selected ISTD**	**Quantification ion ISTD (*m/z*)**
1,5-AG	1846	1521	259	1,5-AG $^{13}C_6$	264
2,4-DHB	1400	1070	219	Levoglucosan $^{13}C_6$	220
Arabinose	1653	1328	307	Arabinose $^{13}C_5$	310
Arabitol	1712	1389	307	Arabitol $^{13}C_5$	310
chiro-Inositol	1950	1630	318	Mannoheptulose $^{13}C_7$	323
Erythritol	1496	1172	307	Erythritol $^{13}C_4$	310
Erythrose	1439	1111	205	Mannose $^{13}C_6$ derivative 1	323
Fructose derivative 2	1646	1551	307	Fructose $^{13}C_6$ derivative 2	310
Fucitol	1777	1454	319	Fructose $^{13}C_6$ derivative 2	310
Fucose derivative 2	1731	1409	277	Fucose $^{13}C_6$ derivative 2	279
Galactitol	1935	1613	331	Galactitol $^{13}C_6$	336
Galactose derivative 2	1907	1560	319	Galactose $^{13}C_6$ derivative 2	323
Galacturonic acid derivative 1	1936	1614	333	Glucuronic acid $^{13}C_6$ derivative 1	337
Glucaric acid	2004	1693	292	Glucose $^{13}C_6$ derivative 2	323
Gluconic acid	1993	1682	333	Gluconic acid $^{13}C_6$	337
Glucose derivative 2	1890	1586	319	Glucose $^{13}C_6$ derivative 2	323
Glucuronic acid derivative 2	1944	1623	333	Glucuronic acid $^{13}C_6$ derivative 2	337
Lactose derivative 1	2673	2344	319	Lactose $^{13}C_{12}$ derivative 1	323
Levoglucosan	1696	1373	217	Levoglucosan $^{13}C_6$	220
Maltose derivative 1	2723	2394	361	Maltose $^{13}C_{12}$ derivative 1	367
Mannitol	1923	1599	331	Mannitol $^{13}C_6$	336
Mannoheptulose	2104	1786	319	Mannoheptulose $^{13}C_7$	323
Mannose derivative 1	1880	1555	319	Mannose $^{13}C_6$ derivative 1	323
myo-Inositol	2089	1774	318	*myo*-Inositol D_6	321
GlcNAc derivative 2	2079	1764	319	GlcNAc $^{13}C_6$ derivative 2	323
Psicose derivative 1	1850	1526	307	Psicose $^{13}C_6$ derivative 1	310
Rhamnose	1710	1386	277	1,5-AG $^{13}C_6$	264
Ribitol	1717	1395	307	Ribitol $^{13}C_5$	310
Ribose	1669	1344	307	Ribose $^{13}C_5$	310
Sedoheptulose derivative 1	2130	1801	319	Mannoheptulose $^{13}C_7$	323
Sorbitol	1930	1607	331	Sorbitol $^{13}C_6$	336
Sucrose	2622	2293	319	Sucrose $^{13}C_{12}$	323
Tagatose derivative 1	1842	1518	307	Tagatose $^{13}C_6$ derivative 1	310
Threitol	1487	1162	307	Arabinose $^{13}C_5$	310
Threonic acid	1548	1225	292	Ribitol $^{13}C_5$	310
Trehalose	2731	2402	361	Trehalose $^{13}C_{12}$	367
Xylitol	1698	1374	307	Xylitol $^{13}C_5$	310
Xylonic acid	1756	1433	292	Levoglucosan $^{13}C_6$	220
Xylose derivative 1	1637	1312	307	Xylose $^{13}C_5$ derivative 1	310
Xylulose	1666	1341	263	Xylulose $^{13}C_5$	266

[a] Generally, all sugar compounds are trimethylsilylated derivatives, and in case of reducing sugars, methoximated. However, for reasons of readability, the underivatized names of the sugar compounds are used. The quantification of reducing sugars was carried out using one derivative.
[b] RI calculated on basis of alkanes.
[c] RI calculated on basis of FAME.
RI: Retention time indices. ISTD: Isotopically labeled internal standards. 1,5-AG: 1,5-Anhydroglucitol. 2,4-DHB: 2,4-Dihydoxbutyric acid. GlcNAc: *N*-Acetylglucosamine. FAME: Fatty acid methyl ester.

Table S15. Quantified sugar compounds in serum, their corresponding RI, as well as selected quantification ion, selected ISTD, and their corresponding quantification ion for the ISTD.

**Sugar compound**[a]	**RI –alkane**[b]	**RI – FAME**[c]	**Quantification ion sugar (*m/z*)**	**Selected ISTD**	**Quantification ion ISTD (*m/z*)**
1,5-AG	1845	1524	259	1,5-AG $^{13}C_6$	264
2,4-DHB	1395	1067	219	Psicose $^{13}C_6$ derivative 2	310
Allose derivative 1	1869	1549	319	Arabitol $^{13}C_5$	310
Arabinonic acid	1770	1449	292	Gluconic acid $^{13}C_6$	337
Arabinose	1649	1326	307	Arabinose $^{13}C_5$	310
Arabitol	1709	1388	307	Arabitol $^{13}C_5$	310
Erythritol	1491	1167	307	Erythritol $^{13}C_4$	310
Erythronic acid	1527	1208	277	Glucuronic acid $^{13}C_6$ derivative 1	337
Fructose derivative 2	1873	1552	277	Fructose $^{13}C_6$ derivative 2	310
Fucose derivative 1	1717	1396	277	Arabitol $^{13}C_5$	310
Galactitol	1934	1615	319	Galactitol $^{13}C_6$	323
Galactose derivative 1	1883	1561	319	Galactose $^{13}C_6$ derivative 1	323
Gluconic acid	1993	1682	333	Gluconic acid $^{13}C_6$	337
Glucose derivative 2	1909	1588	319	Glucose $^{13}C_6$ derivative 2	323
Glucuronic acid derivative 1	1927	1607	333	Glucuronic acid $^{13}C_6$ derivative 1	337
Lactose derivative 1	2673	2347	361	Lactose $^{13}C_{12}$ derivative 1	367
Levoglucosan	1696	1374	217	Levoglucosan $^{13}C_6$	220
Maltose derivative 1	2724	2400	361	Maltose $^{13}C_{12}$ derivative 1	367
Mannitol	1923	1601	319	Mannitol $^{13}C_6$	323
Mannoheptulose	2102	1787	319	Mannoheptulose $^{13}C_7$	323
Mannose derivative 1	1877	1557	291	Mannose $^{13}C_6$ derivative 1	294
myo-Inositol	2089	1775	265	*myo*-Inositol D_6	321
GlcNAc	2074	1761	319	GlcNAc $^{13}C_6$	323
Psicose derivative 1	1847	1526	307	Psicose $^{13}C_6$ derivative 1	310
Ribitol	1715	1395	307	Ribitol $^{13}C_5$	310
Ribonic acid	1756	1435	292	Gluconic acid $^{13}C_6$	337
Ribose	1666	1343	307	Ribose $^{13}C_5$	310
scyllo-Inositol	2025	1715	318	Glucuronic acid $^{13}C_6$ derivative 2	337
Sedoheptulose derivative 2	2136	1814	319	Arabitol $^{13}C_5$	310
Sucrose	2620	2295	361	Sucrose $^{13}C_{12}$	367
Threitol	1481	1158	307	Arabitol $^{13}C_5$	310
Trehalose	2732	2406	361	Trehalose $^{13}C_{12}$	367
Xylitol	1695	1373	307	Xylitol $^{13}C_5$	310
Xylonic acid	1753	1432	292	Gluconic acid $^{13}C_6$	337
Xylose derivative 1	1635	1311	307	Xylose $^{13}C_5$ derivative 1	310
Xylulose	1662	1340	263	Xylulose $^{13}C_5$	266

[a] Generally, all sugar compounds are trimethylsilylated derivatives, and in case of reducing sugars, methoximated. However, for reasons of readability, the underivatized names of the sugar compounds are used. The quantification of reducing sugars was carried out using one derivative.
[b] RI calculated on basis of alkanes.
[c] RI calculated on basis of FAME.
RI: Retention time indices. ISTD: Isotopically labeled internal standards. 1,5-AG: 1,5-Anhydroglucitol. 2,4-DHB: 2,4-Dihydoxbutyric acid. GlcNAc: *N*-Acetylglucosamine. FAME: Fatty acid methyl ester.

S7 Fragment ions

Table S16. Selected known characteristic fragment ions occurring in spectra of trimethylsilylated sugar compounds.

Fragment ion (*m/z*)	Structure	Molecular formula	Remarks/Occurrence
117.03663	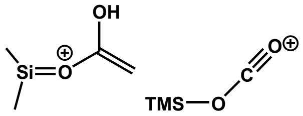	$C_4H_9O_2Si^+$	TMS esters of carboxylic acids [110]
117.07302	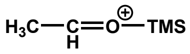	$C_5H_{13}OSi^+$	Deoxy sugars or polyols [110]
129.07302	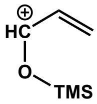	$C_6H_{13}OSi^+$	Unspecific sugar fragment [110]
191.09181	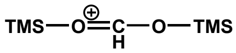	$C_7H_{19}O_2Si_2^+$	Unspecific sugar fragment [110]
203.09181		$C_8H_{19}O_2Si_2^{+}$ [a]	Occurs in 2,3-Dihydroxybutyric acid
205.10746	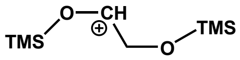	$C_8H_{21}O_2Si_2^+$	Sugars [110]
217.10746	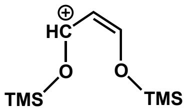	$C_9H_{21}O_2Si_2$	Sugars; formed by TMSOH loss from *m/z* 307 [110]
219.12311	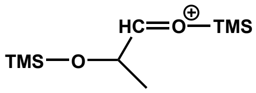	$C_9H_{23}O_2Si_2^+$	Deoxy sugar and polyol
220.0940[b]			Occurs in 2,3-Dihydroxybutyric acid
231.12311	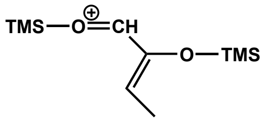	$C_{10}H_{23}O_2Si_2^{+}$ [a]	Occurs in fucitol, derived from *m/z* 321 by elimination of TMSOH
233.10237	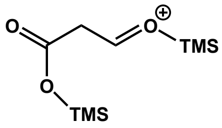	$C_9H_{21}O_3Si_2^+$	2-Deoxy acids like malic acid and related C5/C6 acids [110]
277.14699	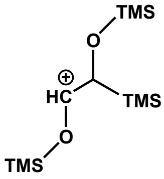	$C_{11}H_{29}O_2Si_3^+$	Derived from *m/z* 307 by expulsion of formaldehyde [111];
292.13408	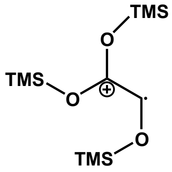	$C_{11}H_{28}O_3Si_3^{\bullet +}$	Organic acids with 2,3-dihydroxy structure; formed by rearrangement [110]
299.07145	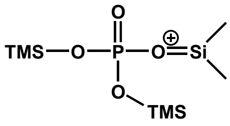	$C_8H_{24}O_4PSi_3^+$	Phosphorylated compounds [110]
305.1419	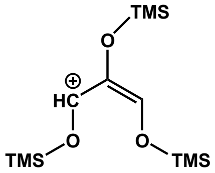	$C_{12}H_{29}O_3Si_3^+$	Prominent with inositols [110]
307.15755	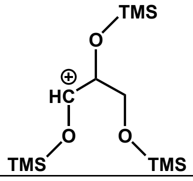	$C_{12}H_{31}O_3Si_3^+$	Sugars; especially C6 ketoses and C5 sugars [110]

Fragment ion (*m/z*)	Structure	Molecular formula	Remarks/Occurrence
315.10275	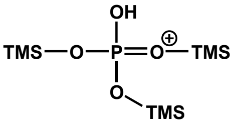	$C_9H_{28}O_4PSi_3^+$	Phosphorylated compounds [110]
319.15755	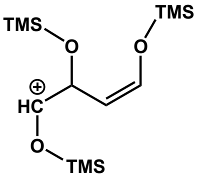	$C_{13}H_{31}O_3Si_3^+$	Methoxime-TMS and TMS derivatives of sugars; derived from *m/z* 409 by elimination of TMSOH [110]
321.1732	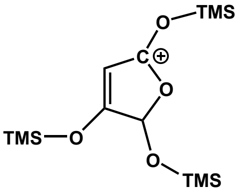	$C_{13}H_{33}O_3Si_2^+$	Deoxy sugars [110]
333.13682	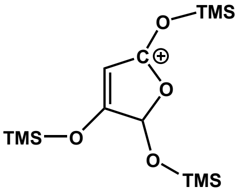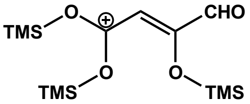	$C_{13}H_{29}O4Si_3^+$	C6 sugar acids (C3-C6); derived from *m/z* 423 by elimination of TMSOH; often combined with *m/z* 292 [110]
357.11332	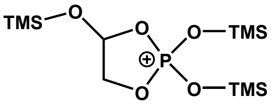	$C_{11}H_{30}O_5PSi_3^+$	Phosphorylated compounds [110]
387.14228	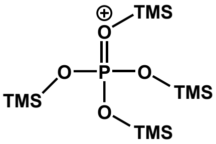	$C_{12}H_{36}O_4PSi_4^+$	Phosphorylated sugars [110]

[a] Proposed structure.
[b] Exact formula not known; *m/z* corresponds to fragment on 2,3-Dihydroxybutyric acid.
TMS: Trimethylsilyl.

S8 Application of the sugaromics method

Table S17. Participant characteristics of the 40 KarMeN volunteers.

Parameter	Value
Sex (female/male)	16/24
Age (year)[a]	47.9±19.1
BMI (kg/m²)[a]	23.6±2.3
Osmolality of urine (mOsm/kg)[a]	406.3±200.0
Concentration of creatinine in urine (mmol/L)[a]	5.8±3.4

[a] Mean ± standard deviation.

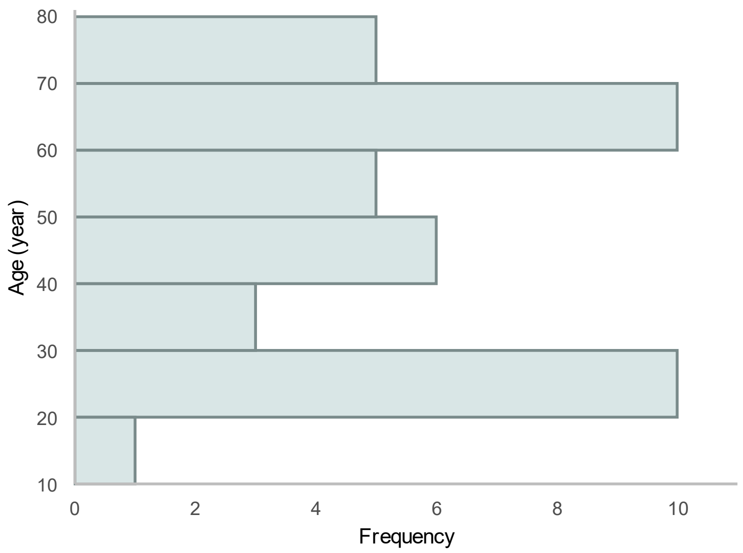

Figure S3. Age distribution of the 40 KarMeN volunteers.

Table S18. Measurement series parameters and conditions for the 40 analyzed urine and serum samples.

Parameter	Urine	Serum
	**Calibration curve**	
	7 levels; additionally, Level 1 was measured 1:2 and 1:10 diluted (at least 5 repetitions)	7 levels (8 repetitions); Extra calibration solutions (5 levels) for glucose (8 repetitions)
	**Accuracy samples**	
	Spiking of the QC sample with two different concentrations of each sugar compound (urine: addition of 30 µL accuracy solution to vial; serum: addition of 20 µL accuracy solution to tube instead of 20 µL MeOH)	
	**Sample preparation**	
	Dilution of the urine to 56 mOsm/kg<br>▼<br>40 µL urine dilution<br>+20 µL ISTD<br>▼<br>Evaporation<br>▼<br>2-Step-derivatization	20 µL serum + 10 µL ISTD<br>+ 220 µL cold MeOH<br>▼<br>Centrifugation:<br>10 min, 16100 × g, 4 °C<br>▼<br>Evaporation of 160 µL supernatant<br>▼<br>2-Step-derivatization
MeOX conditions	15 µL, 1 h at 40 °C, 1000 rpm	25 µL, 0.5 h at 70 °C, 600 rpm
MSTFA conditions	50 µL, 1.5 h at 75 °C, 1000 rpm	50 µL, 1.5 h at 75 °C, 800 rpm
	**GC×GC-MS analysis**	
Colum combination	^{1}D: Rxi-5 SilMS (Restek, Germany; 40(+5) m × 0.18 mm ID × 0.36 µm film thickness)<br>^{2}D: V17ms (Agilent, USA; 2.1 m (thereof 0.9 m as modulator loop and 1 m for ^{2}D separation) × 0.15 mm ID × 0.15 µm film thickness)	
Linear velocity	25 cm/s	25 cm/s
Initial head pressures	300.3 kPa	272.4 kPa
Injection mode	programmed temperature vaporization:<br>90 °C-280 °C; 60 °C/s	Hot injection at 280 °C
Injection volume	1 µL	1 µL
Split ratio	1:5 (after 1min 1:40, after 20min 1:10)	1:5 (after 1min 1:50, after 20min 1:10)
Oven temperature program	140 °C→ 1.25 °C/min→ 175 °C→ 1.5 °C/min→ 210 °C→ 8 °C/min→ 255°C→ 3 °C/min→ 300 °C (hold for 3.04 min);<br>total run time: 75 min	100 °C→ 12 °C/min→ 150 °C→ 1.75 °C/min→ 220 °C→ 8 °C/min→ 280 °C→ 3 °C/min→ 310 °C→ 25 °C/min→ 330 °C<br>(hold for 6.53 min);<br>total run time: 69 min
Modulation time	2.4 sec	2.1 sec
Ion source temperature	200 °C	250 °C
Interface temperature	300 °C	300 °C
MS *m/z* and time ranges	7-11 min; *m/z*: 210-324<br>11-34 min; *m/z*: 203-317<br>34-68.4 min; *m/z*: 230-344<br>68.4-74.99 min; m/z: 306-420	8-27.3 min; *m/z*: 203-317<br>27.3-55.2 min; *m/z*: 230-344<br>55.2-68.9 min; *m/z*: 306-420

ISTD: Isotopically labeled internal standards; MeOH: Methanol. MeOX: Methoxylamine. MSTFA: *N*-Methyl-*N*-trimethylsilyltrifluoracetamide.

S9 Comparison with reference method

S9.1 High performance liquid chromatography coupled with a refractive index detector (HPLC-RI)[1] method

Soluble sugars (sucrose, fructose and glucose) were determined by HPLC-RI (Agilent 1100 series HPLC, Agilent, Germany). The chromatographic separation was performed on a Reprosil polyamine column (Dr. Maisch, Germany, 5 µm, 250x4.6 mm) at a column temperature of 35 °C with acetonitrile (ACN):H_2O (70:30) and a flow rate of 0.9 mL/min. Standards were purchased from Sigma-Aldrich, USA.

Aliquots of fruit or vegetable juice were stirred in a 100 mL volumetric flask with H_2O (60 °C) for 30 min, filled up to the mark after cooling, and subsequently, filtered through a pleated filter. An aliquot was then diluted with three parts by volume of ACN, which was then filtered through an ultrafilter with a 0.2 µm pore size. 20 µL of the filtrate was

[1] When discussing HPLC, “RI” refers to the refractive index detector (not the retention time index).

injected into the HPLC. The selection of the sample weight (and, consequently, the dilution factor) was within the range of 1:10 to 1:100, depending on the sugar content of the given juice. It is important to note that high total soluble sugar concentrations lead to phase separation during the subsequent dilution with acetonitrile[112], which was to be avoided. Nevertheless, the dilution was initially set as low as possible in order to be able to capture any small amounts of maltose. In instances where the concentrations of individual sugars present vary strongly, two distinct dilutions were used to ensure that the respective signals lay within the linear detector range.

The linear quantification range of the method lies between 0.1 mg/mL – 3 mg/mL (lowest and highest calibration solution). The LOQ of the method is 0.1 – 1 g sugar/100 g juice, depending on the sample quantity and dilution used. The precision was determined by the fivefold measurement of cloudy apple juice and RSD% lies within 1.1-2.7 for the measured sugars.

The accuracy was determined by spiking apple juice and sauerkraut juice. The results were determined for two different dilutions (see Supporting Information Table S15)

Table S19. Accuracy for the HPLC-Method.

		Accuracy [%]			
	Dilution	Fructose	Glucose	Sucrose	Maltose
Apple juice	1:10/1:50	out of range/99	95/96	86/102	96*/97*
Sauerkraut juice	1:10/1:25	82/103*	73/95*	107*/98*	98*/100*

*For samples with very low concentrations and/or no detectable signal, calculation via x-axis interception was not feasible, accuracy was then calculated in relation to calibration curve

S9.2 Sample preparation of fruit juice, purée and oat drink for sugaromics analysis

For the sugaromics analysis, juices and purées were diluted, while oat drink was extracted. In detail, for the analytic of the juices (apple, aronia, sauerkraut, pear) and purées (peach, apricot) 50 mg material were added in a 50 mL volumetric flask and filled up with water to the mark. Then, an aliquot was transferred into a vial and ISTD solution was added. For the sample preparation of oat drink, 50 mg were extracted twice, first with MeOH and with MeOH/H_2O (1:1) afterward. Aliquots of the supernatant were transferred into a vial and ISTD solution was added. Further preparation was carried out as described in the main manuscript Figure 1 for the urine analysis.

S9.3 Results of the analysis using the reference and sugaromics method

Table S20. Measured and rounded concentration of fructose, glucose and sucrose in fruit juices and plant drinks using the reference (HPLC-RI) and sugaromics (GC×GC-MS) method, as well as the percentage deviation of the measured values.

	**Fructose**			**Glucose**			**Sucrose**		
	**Concentration [mmol/100g]**		**Deviation [%]**	**Concentration [mmol/100g]**		**Deviation [%]**	**Concentration [mmol/100g]**		**Deviation [%]**
**Juice/ purée/ drink**	**HPLC-RI**	**Sugaromics**		**HPLC-RI**	**Sugaromics**		**HPLC-RI**	**Sugaromics**	
Apple	31.6	30.8	-2.4	9.4	11.5	22.8	5.9	3.4	-42.6
Apricot	8.9	9.3	4.7	12.7	12.7	-0.5	8.9	9.1	2.7
Aronia	16.7	17.0	2.1	17.3	19.5	12.8	<LLOQ	0.004	--
Pear	32.7	33.1	1.1	8.9	9.4	5.9	2.6	2.7	3.8
Peach	12.2	12.3	0.7	10.6	10.2	-3.9	11.3	11.3	-0.1
Sauerkraut	0.6	1.0	60.9	4.4	4.8	8.8	<LLOQ	0.002	--
Oat milk	0.03	0.02	-51.7	0.12	0.10	-21.5	0.3	0.3	10.5

LLOQ: Lower limit of quantification.

S10 Carryover

Table S21. Evaluation of the carryover measurements. Measured mean heights in the blank samples (n=3) after injection of a calibration level.

	**Urine**			**Serum**								
	**Levoglucosan**			**Fructose**			**Sucrose**			**Levoglucosan**		
	c[μmol/L][a]	Height[b]	n[c]	c[μmol/L][a]	Height[b]	n[c]	c[μmol/L][a]	Height[b]	n[c]	c[μmol/L][a]	Height[b]	n[c]
	0.01	86931	2	0.1	40338	2	0.02	111398	3	0.02	17990	3
	0.1	62478	2	2.5	37016	2	0.1	126970	2	0.075	23724	3
	1	49205	1	10	38282	2	0.5	94423	3	0.125	20121	2
	4	63093	2	25	41494	2	1	89515	3	0.5	19787	3
	7	54362	2	50	41284	2	2.5	105252	3	1	11587	2
	10	58614	2	100	---	0	5	60428	2	2	17398	2
	15	57517	2	200	50056	1	7.5	84809	3	5	18867	3
	30	48018	3									
Mean height of all blanks		60027			41412			96114			18496	
RSD% of all blanks [%]		54.2			15.2			35.7			30.5	
Percentage of the mean peak height of all blanks to the LLOQ [%]		46[e]			32			12			35	
Percentage of the mean peak height of all blanks to the next higher calibration level [%][d]		8[f]			24			---			23	

[a] Concentration of the measured calibration level before blank sample.
[b] Mean height signal in blank samples.
[c] Number of blank samples where peaks were observed.
[d] Calculated if percentage of the mean peak height of all blanks to the LLOQ > 25%
[e] Peak heights of a 0.1 μmol/L solution were used.
[f] Peak heights of a 1 μmol/L solution were used.
RSD%: Relative standard deviation. LLOQ: Lower limit of quantification.

S11 Correlation between osmolality and creatinine

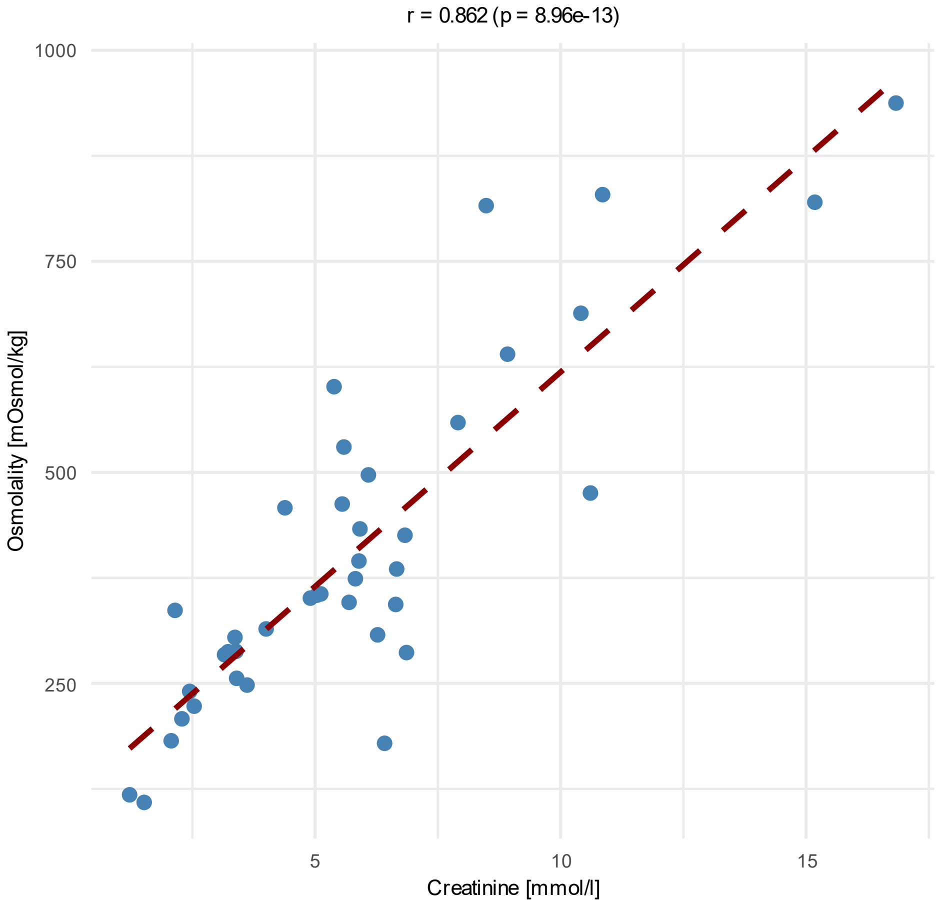

Figure S4. Correlation between osmolality and creatinine in 40 urine samples of the application study.

References

(1) Shimizu, H.; Shouzu, A.; Nishikawa, M.; Omoto, S.; Hayakawa, T.; Miyake, Y.; Yonemoto, T.; Inada, M. Serum Concentration and Renal Handling of 1,5-Anhydro-D-Glucitol in Patients with Chronic Renal Failure. *Ann. Clin. Biochem.* **1999**, *36* (6), 749-754.

(2) Ren, J.; Ma, Y.; Ma, M.; Ding, J.; Jiang, J.; Zheng, X.; Han, X. Rapid ultra-performance liquid chromatography-tandem mass spectrometry method for the simultaneous determination of three characteristic urinary saccharide metabolites in patients with glycogen storage diseases (type Ⅰb and Ⅱ). *J. Chromatogr. B.* **2023**, *1229*, 123900.

(3) Bouatra, S.; Aziat, F.; Mandal, R.; Guo, A. C.; Wilson, M. R.; Knox, C.; Bjorndahl, T. C.; Krishnamurthy, R.; Saleem, F.; Liu, P.; et al. The Human Urine Metabolome. *PLoS ONE* **2013**, *8* (9), e73076.

(4) Jansen, G.; Muskiet, F. A. J.; Schierbeek, H.; Berger, R.; van der Slik, W. Capillary gas chromatographic profiling of urinary, plasma and erythrocyte sugars and polyols as their trimethylsilyl derivatives, preceded by a simple and rapid prepurification method. *Clin. Chim. Acta* **1986**, *157* (3), 277-293.

(5) Andersen, J. R.; Bukhave, K.; Højgaard, L.; Rasmussen, H. S.; Hermansen, N.; Worning, H.; Krag, E. Decomposition of Wheat Bran and Ispaghula Husk in the Stomach and the Small Intestine of Healthy Men. *J. Nutr.* **1988**, *118* (3), 326-331.

(6) Ge, S.-l.; Wang, H.; Wang, Z.-F.; Cheng, S.; Wang, Q.-J.; He, P.-G.; Fang, Y.-Z. Sensitive measurement of polyols in urine by capillary zone electrophoresis coupled with amperometric detection using on-column complexation with borate. *J. Chromatogr. B* **2013**, *915-916*, 39-45.

(7) Sṕencer, N. Ion exchange chromatograhy of polyols. *J. Chromatogr. A* **1967**, *30*, 566-571.
(8) Pitkänen, E. The serum polyol pattern and the urinary polyol excretion in diabetic and in uremic patients. *Clin. Chim. Acta* **1972**, *38* (1), 221-230.
(9) Campbell, W. W.; Ostlund, J. R. E.; Joseph, L. J.; Farrell, P. A.; Evans, W. J. Relationships of Plasma C-Peptide and Gender to the Urinary Excretion of Inositols in Older People. *Horm. Metab. Res.* **2001**, *33* (1), 44-51.
(10) Jung, T.-S.; Hahm, J.-R.; Kim, J.-J.; Jung, J.-H.; Kang, M.-Y.; Moon, S.-W.; Lee, K.-W.; Kim, H.-C.; Lee, J.-D.; Kim, J.-H.; et al. Determination of Urinary Myo-/Chiro-Inositol Ratios from Korean Diabetes Patients. *Yonsei Med. J.* **2005**, *46* (4), 532.
(11) Suzuki, S.; Kawasaki, H.; Satoh, Y.; Ohtomo, M.; Hirai, M.; Hirai, A.; Hirai, S.; Onoda, M.; Matsumoto, M.; Hinokio, Y.; et al. Urinary chiro-Inositol Excretion is an Index Marker of Insulin Sensitivity in Japanese Type II Diabetes. *Diabetes Care* **1994**, *17* (12), 1465-1468.
(12) Hassanzadeh, J.; Al Lawati, H. A. J.; Bagheri, N. On paper synthesis of multifunctional CeO2 nanoparticles@Fe-MOF composite as a multi-enzyme cascade platform for multiplex colorimetric detection of glucose, fructose, sucrose, and maltose. *Biosens. Bioelectron.* **2022**, *207*, 114184.
(13) Tasevska, N.; Runswick, S. A.; McTaggart, A.; Bingham, S. A. Urinary sucrose and fructose as biomarkers for sugar consumption. *Cancer Epidemiol., Biomarkers Prev.* **2005**, *14* (5), 1287-1294.
(14) Song, X.; Navarro, S. L.; Diep, P.; Thomas, W. K.; Razmpoosh, E. C.; Schwarz, Y.; Wang, C.-Y.; Kratz, M.; Neuhouser, M. L.; Lampe, J. W. Comparison and validation of 2 analytical methods for measurement of urinary sucrose and fructose excretion. *Nutr. Res.* **2013**, *33* (9), 696-703.
(15) Diepeveen-de Bruin, M.; Maho, W.; Buso, M. E. C.; Naomi, N. D.; Brouwer-Brolsma, E. M.; Feskens, E. J. M.; Balvers, M. G. J. Development and validation of a UPLC-MS/MS method for the quantification of sugars and non-nutritive sweeteners in human urine. *J. Chromatogr. B* **2023**, *1225*, 123741.
(16) Buso, M. E. C.; Boshuizen, H. C.; Naomi, N. D.; Maho, W.; Diepeveen-de Bruin, M.; Balvers, M. G. J.; de Vries, J. H. M.; Harrold, J. A.; Halford, J. C. G.; Raben, A.; et al. Relative validity of habitual sugar and low/no-calorie sweetener consumption assessed by food frequency questionnaire, multiple 24-h dietary recalls and urinary biomarkers: an observational study within the SWEET project. *Am. J. Clin. Nutr.* **2024**, *119* (2), 546-559.
(17) Tasevska, N.; Midthune, D.; Potischman, N.; Subar, A. F.; Cross, A. J.; Bingham, S. A.; Schatzkin, A.; Kipnis, V. Use of the Predictive Sugars Biomarker to Evaluate Self-Reported Total Sugars Intake in the Observing Protein and Energy Nutrition (OPEN) Study. *Cancer Epidemiol., Biomarkers Prev.* **2011**, *20* (3), 490-500.
(18) Freedman, L. S.; Kipnis, V.; Midthune, D.; Commins, J.; Barrett, B.; Sagi-Kiss, V.; Palma-Duran, S. A.; Johnston, C. S.; O'Brien, D. M.; Tasevska, N. Establishing 24-Hour Urinary Sucrose Plus Fructose as a Predictive Biomarker for Total Sugars Intake. *Cancer Epidemiol., Biomarkers Prev.* **2022**, *31* (6), 1227-1232.
(19) Yamauchi, M.; Kimura, K.; Maezawa, Y.; Ohata, M.; Mizuhara, Y.; Hirakawa, J.; Nakajima, H.; Toda, G. Urinary level of L-fucose as a marker of alcoholic liver disease. *Alcohol.: Clin. Exp. Res.* **1993**, *17* (2), 268-271.
(20) Yamauchi, M.; Nishikawa, F.; Kimura, K.; Maezawa, Y.; Ohata, M.; Toda, G. Urinaryl-fucose: A new marker for the diagnosis of chronic liver disease. *Hepatol. Res.* **1993**, *1* (4), 222-227.
(21) Schadewaldt, P.; Kamalanathan, L.; Hammen, H.-W.; Wendel, U. Age dependence of endogenous galactose formation in Q188R homozygous galactosemic patients. *Molecular Genetics and Metabolism* **2004**, *81* (1), 31-44.

(22) Schadewaldt, P.; Hammen, H.-W.; Stolpmann, S.; Kamalanathan, L.; Wendel, U. Galactonate determination in urine by stable isotope dilution gas chromatography–mass spectrometry. *J. Chromatogr. B* **2004**, *801* (2), 249-255.
(23) Colombi, A.; Maroni, M.; Antonini, C.; Fait, A.; Zocchetti, C.; Foà, V. Influence of sex, age, and smoking habits on the urinary excretion of d-glucaric acid. *Clin. Chim. Acta* **1983**, *128* (2), 349-358.
(24) Ayotte, P.; Dewailly, É.; Lambert, G. H.; Perkins, S. L.; Poon, R.; Feeley, M.; Larochelle, C.; Pereg, D. Biomarker Measurements in a Coastal Fish-Eating Population Environmentally Exposed to Organochlorines. *Environ. Health Perspect.* **2005**, *113* (10), 1318-1324.
(25) Ohnhaus, E. E.; Park, B. K. Measurement of urinary 6-β-hydroxycortisol excretion as an in vivo parameter in the clinical assessment of the microsomal enzyme-inducing capacity of antipyrine, phenobarbitone and rifampicin. *Eur. J. Clin. Pharmacol.* **1979**, *15* (2), 139-145.
(26) Sandstad, O.; Osnes, T.; Skar, V.; Osnes, M. Urinary D-glucaric acid, a marker substance for microsomal enzyme induction. Methodological aspects, responses to alcohol and findings in workers exposed to toluene. *Scand. J. Clin. Lab. Invest.* **1993**, *53* (4), 327-333.
(27) Saady, J. J.; Blanke, R. V. Measurement of Glucuronic Acid Metabolites by High-Resolution Gas Chromatography. *Journal of Chromatographic Science* **1990**, *28* (6), 282-287.
(28) Apostoli, P.; Mangili, A.; Carasi, S.; Manno, M. Relationship between PCBs in blood and d-glucaric acid in urine. *Toxicol. Lett.* **2003**, *144* (1), 17-26.
(29) Tutor, J. C.; Lopez-Urrutia, A.; Fernandez, M. P. Urinary Excretion of D-Glucaric Acid in Porphyria Cutanea Tarda. *Clin. Chem. Lab. Med.* **1987**, *25* (12).
(30) Bauer, S.; Störmer, E.; Kerb, R.; Johne, A.; Brockmöller, J.; Roots, I. Differential effects of Saint John's Wort (hypericum perforatum) on the urinary excretion of D -glucaric acid and 6β-hydroxycortisol in healthy volunteers. *Eur. J. Clin. Pharmacol.* **2002**, *58* (9), 581-585.
(31) Sandle, L. N.; Braganza, J. M. An evaluation of the low-pH enzymatic assay of urinary d-glucaric acid, and its use as a marker of enzyme induction in exocrine pancreatic disease. *Clin. Chim. Acta* **1987**, *162* (3), 245-256.
(32) Marsh, C. A. An enzymatic determination of d-glucaric acid by conversion to pyruvate. *Anal. Biochem.* **1985**, *145* (2), 266-272.
(33) Sotaniemi, E. A.; Pelkonen, R. O.; Puukka, M. Measurement of hepatic drug-metabolizing enxyme activity in man. Comparison of three different assays. *Eur. J. Clin. Pharmacol.* **1980**, *17* (4), 267-274.
(34) Weiersmüller, A.; Colombo, J. P.; Bircher, J. The influence of prednisolone on hepatic function in normal subjects. *Am. J. Dig. Dis.* **1977**, *22* (5), 424-428.
(35) Shaykhutdinov, R. A.; Macinnis, G. D.; Dowlatabadi, R.; Weljie, A. M.; Vogel, H. J. Quantitative analysis of metabolite concentrations in human urine samples using 13C{1H} NMR spectroscopy. *Metabolomics* **2009**, *5* (3), 307-317.
(36) Li, T.; Ihanus, A.; Ohukainen, P.; Järvelin, M. R.; Kettunen, J.; Mäkinen, V. P.; Tynkkynen, T.; Ala-Korpela, M. There is always glucose in normal urine: unspecific excretion associated with serum glucose and glomerular filtration rate. *Int. J. Epidemiol.* **2022**, *51* (6), 2022-2025.
(37) Kawasaki, T.; Akanuma, H.; Yamanouchi, T. Increased Fructose Concentrations in Blood and Urine in Patients With Diabetes. *Diabetes Care* **2002**, *25* (2), 353-357.

(38) Kim, S. R.; Lee, Y.-H.; Lee, S.-G.; Lee, S. H.; Kang, E. S.; Cha, B.-S.; Lee, H. C.; Kim, J.-H.; Lee, B.-W. Morning Spot Urine Glucose-to-Creatinine Ratios Predict Overnight Urinary Glucose Excretion in Patients With Type 2 Diabetes. *Annals of Laboratory Medicine* **2017**, *37* (1), 9-17.

(39) Muting, D.; Reikowski, J.; STUMM, D. Untersuchungen über die Entgiftungsfunktion des menschlichen Organismus. 1. Die Ausscheidung von Glucuronsäure, freien und gebundenen Sulfaten im 24-Std-Urin von 120 gesunden Menschen. *Z. Klin. Med.* **1962**, *157* (4), 391-404.

(40) Keltanen, T. N.; Heikman, P. K.; Muhonen, L. H.; Gunnar, T. O.; Ojanperä, I. A. Enzymatic assay for urine lactose in the assessment of recent intravenous abuse of buprenorphine. *Drug Test. Anal.* **2019**, *11* (9), 1412-1418.

(41) Moshammer, H.; Weiss, S.; Neuberger, M. Woodsmoke marker levoglucosan: Kinetics in a self-experiment. *International Journal of Occupational Medicine and Environmental Health* **2012**, *25* (2).

(42) Hinwood, A. L.; Trout, M.; Murby, J.; Barton, C.; Symons, B. Assessing urinary levoglucosan and methoxyphenols as biomarkers for use in woodsmoke exposure studies. *Sci. Total Environ.* **2008**, *402* (1), 139-146.

(43) Mazzarino, M.; Fiacco, I.; De La Torre, X.; Botrè, F. A rapid analytical method for the detection of plasma volume expanders and mannitol based on the urinary saccharides and polyalcohols profile. *Drug Test. Anal.* **2011**, *3* (11-12), 896-905.

(44) Guddat, S.; Thevis, M.; Schänzer, W. Identification and Quantification of the Osmodiuretic Mannitol in Urine for Sports Drug Testing Using Gas Chromatography-Mass Spectrometry. *Eur. J. Mass Spectrom.* **2008**, *14* (3), 127-133.

(45) Wamelink, M. M.; Smith, D. E.; Jansen, E. E.; Verhoeven, N. M.; Struys, E. A.; Jakobs, C. Detection of transaldolase deficiency by quantification of novel seven-carbon chain carbohydrate biomarkers in urine. *J. Inherited Metab. Dis.* **2007**, *30* (5), 735-742.

(46) Kouzuma, T.; Takahashi, M.; Endoh, T.; Kaneko, R.; Ura, N.; Shimamoto, K.; Watanabe, N. An enzymatic cycling method for the measurement of myo-inositol in biological samples. *Clin. Chim. Acta* **2001**, *312* (1), 143-151.

(47) Perelló, J.; Isern, B.; Costa-Bauzá, A.; Grases, F. Determination of myo-inositol in biological samples by liquid chromatography–mass spectrometry. *J. Chromatogr. B* **2004**, *802* (2), 367-370.

(48) Yamakoshi, M.; Takahashi, M.; Kouzuma, T.; Imamura, S.; Tsuboi, I.; Kawazu, S.; Yamagata, F.; Tominaga, M.; Noritake, M. Determination of urinary myo-inositol concentration by an improved enzymatic cycling method using myo-inositol dehydrogenase from Flavobacterium sp. *Clin. Chim. Acta* **2003**, *328* (1), 163-171.

(49) Yoshii, H.; Uchino, H.; Ohmura, C.; Watanabe, K.; Tanaka, Y.; Kawamori, R. Clinical usefulness of measuring urinary polyol excretion by gas-chromatography/mass-spectrometry in type 2 diabetes to assess polyol pathway activity. *Diabetes Res. Clin. Pract.* **2001**, *51* (2), 115-123.

(50) Zhu, X.; Wei, Y.; He, Y.; He, R.; Li, J. Urine D-ribose levels correlate with cognitive function in community-dwelling older adults. *BMC Geriatr.* **2022**, *22* (1).

(51) Monnard, I.; Bénet, T.; Jenni, R.; Austin, S.; Silva-Zolezzi, I.; Godin, J.-P. Plasma and urinary inositol isomer profiles measured by UHPLC-MS/MS reveal differences in scyllo-inositol levels between non-pregnant and pregnant women. *Anal. Bioanal. Chem.* **2020**, *412* (28), 7871-7880.

(52) Wamelink, M. M. C.; Struys, E. A.; Jansen, E. E. W.; Levtchenko, E. N.; Zijlstra, F. S. M.; Engelke, U.; Blom, H. J.; Jakobs, C.; Wevers, R. A. Sedoheptulokinase deficiency due to a 57-kb deletion in cystinosis patients causes urinary accumulation of sedoheptulose: elucidation of the CARKL gene. *Hum. Mutat.* **2008**, *29* (4), 532-536.

(53) McNamara, A. E.; Walton, J.; Flynn, A.; Nugent, A. P.; McNulty, B. A.; Brennan, L. The Potential of Multi-Biomarker Panels in Nutrition Research: Total Fruit Intake as an Example. *Front. Nutr.* **2021**, *7*, 577720.
(54) Ciucanu, I.; Pilat, L.; Ciucanu, C. I.; Şişu, E. Determination of Neutral Monosaccharides as Per-O-methylated Derivatives Directly from a Drop of Whole Blood by Gas Chromatography–Mass Spectrometry. *Anal. Chem.* **2015**, *87* (21), 10856-10861.
(55) Phillipou, G.; James, S. K.; Frith, R. G.; Farrant, R. K.; Phillips, P. J. Enzymatic quantification of 1,5-anhydro-D-glucitol: evaluation and clinical application. *Clin. Chem.* **1994**, *40* (7 Pt 1), 1322-1326.
(56) Shetty, H. U.; Holloway, H. W.; Rapoport, S. I. Capillary Gas Chromatography Combined with Ion Trap Detection for Quantitative Profiling of Polyols in Cerebrospinal Fluid and Plasma. *Anal. Biochem.* **1995**, *224* (1), 279-285.
(57) Kusmierz, J.; DeGeorge, J. D.; Sweeney, D.; May, C.; Rapoport, S. I. Quantitative analysis of polyols in human plasma and cerebrospinal fluid. *J. Chromatogr. B:Biomed. Sci. Appl.* **1989**, *497*, 39-48.
(58) Skupien, J.; Gorczynska-Kosiorz, S.; Klupa, T.; Wanic, K.; Button, E. A.; Sieradzki, J.; Malecki, M. T. Clinical Application of 1,5-Anhydroglucitol Measurements in Patients with Hepatocyte Nuclear Factor-1α Maturity-Onset Diabetes of the Young. *Diabetes Care* **2008**, *31* (8), 1496-1501.
(59) Yamanouchi, T.; Akanuma, H.; Nakamura, T.; Akaoka, I.; Akanuma, Y. Reduction of plasma 1,5-anhydroglucitol (1-deoxyglucose) concentration in diabetic patients. *Diabetologia* **1988**, *31* (1), 41-45.
(60) Inada, S.; Koga, M. Alcohol consumption reduces HbA1c and glycated albumin concentrations but not 1,5-anhydroglucitol. *Ann. Clin. Biochem.* **2017**, *54* (6), 631-635.
(61) Hamafuji, T.; Tsugawa, W.; Sode, K. Clinical application of the serum 1,5-anhydroglucitol assay method using glucose 3-dehydrogenase. *J. Clin. Lab. Anal.* **2002**, *16* (6), 299-303.
(62) Koga, M.; Murai, J.; Saito, H.; Mukai, M.; Kasayama, S.; Moriwaki, Y.; Yamamoto, T. Close relationship between serum concentrations of 1,5-anhydroglucitol and uric acid in non-diabetic male subjects implies common renal transport system. *Clin. Chim. Acta* **2009**, *410* (1), 70-73.
(63) Selvin, E.; Warren, B.; He, X.; Sacks, D. B.; Saenger, A. K. Establishment of Community-Based Reference Intervals for Fructosamine, Glycated Albumin, and 1,5-Anhydroglucitol. *Clin. Chem. (Oxford, U. K.)* **2018**, *64* (5), 843-850.
(64) Gobor, L. C.; Volanski, W.; Boritza, K. C.; Souza, S. W. D.; Anghebem, M. I. A. M. I.; Picheth, G.; Rego, F. G. D. M. Evaluation of 1,5-Anhydroglucitol as a Biomarker for Type 2 Diabetes Mellitus in Patients without Overt Nephropathy. *Braz. J. Pharm. Sci.* **2021**, *57*.
(65) Nowatzke, W.; Sarno, M. J.; Birch, N. C.; Stickle, D. F.; Eden, T.; Cole, T. G. Evaluation of an assay for serum 1,5-anhydroglucitol (GlycoMark™) and determination of reference intervals on the Hitachi 917 analyzer. *Clin. Chim. Acta* **2004**, *350* (1), 201-209.
(66) Selvin, E.; Steffes, M. W.; Ballantyne, C. M.; Hoogeveen, R. C.; Coresh, J.; Brancati, F. L. Racial Differences in Glycemic Markers: A Cross-sectional Analysis of Community-Based Data. *Ann. Intern. Med.* **2011**, *154* (5), 303-309.
(67) Chen, C.; Wang, X.; Tan, Y.; Yang, J.; Yuan, Y.; Chen, J.; Guo, H.; Wang, B.; Sun, Z.; Wang, Y. Reference intervals for serum 1,5-anhydroglucitol of a population with normal glucose tolerance in Jiangsu Province. *J. Diabetes* **2020**, *12* (6), 447-454.

(68) Martins, B. R.; Gomes, L. C.; Boritza, K. C.; Anghebem-Oliveira, M. I.; Souza, E. M.; França, S. N.; Picheth, G.; Rego, F. G. M. Serum 1,5-Anhydroglucitol Concentration as a Biomarker for Type 1 Diabetes in Adults and Children. *Clin Lab* **2019**, *65* (9).

(69) Katayama, M.; Matsuda, Y.; Kobayashi, K.; Kaneko, S.; Ishikawa, H. Simultaneous determination of glucose, 1,5-anhydrod-glucitol and related sugar alcohols in serum by high-performance liquid chromatography with benzoic acid derivatization. *Biomed. Chromatogr.* **2006**, *20* (5), 440-445.

(70) Date, J. W. The Excretion of Lactose and Some Monosaccharides During Pregnancy and Lactation. *Scand. J. Clin. Lab. Invest.* **1964**, *16* (6), 589-596.

(71) Yeo, S. F.; Zhang, Y.; Schafer, D.; Campbell, S.; Wong, B. A rapid, automated enzymatic fluorometric assay for determination of D-arabinitol in serum. *J. Clin. Microbiol.* **2000**, *38* (4), 1439-1443.

(72) Switchenko, A. C.; Miyada, C. G.; Goodman, T. C.; Walsh, T. J.; Wong, B.; Becker, M. J.; Ullman, E. F. An automated enzymatic method for measurement of D-arabinitol, a metabolite of pathogenic Candida species. *J. Clin. Microbiol.* **1994**, *32* (1), 92-97.

(73) Wong, B.; Castellanos, M. Enantioselective measurement of the Candida metabolite d-arabinitol in human serum using multi-dimensional gas chromatography and a new chiral phase. *J. Chromatogr. B:Biomed. Sci. Appl.* **1989**, *495*, 21-30.

(74) Cheng, Y.; Li, L.; Zhu, B.; Liu, F.; Wang, Y.; Gu, X.; Yan, C. Expanded metabolomics approach to profiling endogenous carbohydrates in the serum of ovarian cancer patients. *J. Sep. Sci.* **2016**, *39* (2), 316-323.

(75) Roboz, J.; Suzuki, R.; Holland, J. F. Quantification of arabinitol in serum by selected ion monitoring as a diagnostic technique in invasive candidiasis. *J. Clin. Microbiol.* **1980**, *12* (4), 594-601.

(76) Roboz, J.; Kappatos, D. C.; Greaves, J.; Holland, J. F. Determination of polyols in serum by selected ion monitoring. *Clin Chem* **1984**, *30* (10), 1611-1615.

(77) Wong, B.; Brauer, K. L. Enantioselective measurement of fungal D-arabinitol in the sera of normal adults and patients with candidiasis. *J. Clin. Microbiol.* **1988**, *26* (9), 1670-1674.

(78) Deacon, A. G. Estimations of serum arabinitol for diagnosing invasive candidosis. *J. Clin. Pathol.* **1986**, *39* (8), 842-850.

(79) Wang, X.; Lu, L.; Peng, H.; Li, T.; Long, Q.; Guan, L.; Xia, X.; Li, X.; Wang, M. A rapid and validated GC-MS/MS method for simultaneous quantification of serum Myo- and D-chiro-inositol isomers. *J. Chromatogr. A* **2024**, *1732*, 465246.

(80) Le, M. T.; Frye, R. F.; Rivard, C. J.; Cheng, J.; McFann, K. K.; Segal, M. S.; Johnson, R. J.; Johnson, J. A. Effects of high-fructose corn syrup and sucrose on the pharmacokinetics of fructose and acute metabolic and hemodynamic responses in healthy subjects. *Metabolism* **2012**, *61* (5), 641-651.

(81) Bian, C.; Wang, Y.; Li, J.; Gao, J.; Luan, Z.; Cui, X.; Ren, H. Endogenous fructose is correlated with urinary albumin creatinine ratios and uric acid in type 2 diabetes mellitus. *Diabetes Res. Clin. Pract.* **2021**, *179*, 109034.

(82) Han, B.; Park, J. W.; Kang, M.; Kim, B.; Jeong, J.-s.; Kwon, O.-S.; Son, J. Simultaneous analysis of monosaccharides using ultra high performance liquid chromatography-high resolution mass spectrometry without derivatization for validation of certified reference materials. *J. Chromatogr. B* **2020**, *1160*, 122370.

(83) Macdonald, I.; Keyser, A.; Pacy, D. Some effects, in man, of varying the load of glucose, sucrose, fructose, or sorbitol on various metabolites in blood12. *Am. J. Clin. Nutr.* **1978**, *31* (8), 1305-1311.

(84) Schadewaldt, P.; Hammen, H. W.; Loganathan, K.; Bodner-Leidecker, A.; Wendel, U. Analysis of concentration and (13)C enrichment of D-galactose in human plasma. *Clin. Chem.* **2000**, *46* (5), 612-619.
(85) Pimentel, G.; Burton, K. J.; Rosikiewicz, M.; Freiburghaus, C.; Von Ah, U.; Münger, L. H.; Pralong, F. P.; Vionnet, N.; Greub, G.; Badertscher, R.; et al. Blood lactose after dairy product intake in healthy men. *Br. J. Nutr.* **2017**, *118* (12), 1070-1077.
(86) Blumenthal, H. J.; Lucuta, V. L.; Blumenthal, D. C. Specific enzymatic assay for d-glucarate in human serum. *Anal. Biochem.* **1990**, *185* (2), 286-293.
(87) Asthana, C.; Peterson, G. M.; Shastri, M. D.; Patel, R. P. A novel and sensitive HILIC-CAD method for glucosamine quantification in plasma and its application to a human pharmacokinetic study. *J. Pharm. Biomed. Anal.* **2020**, *178*, 112954.
(88) Roda, A.; Sabatini, L.; Barbieri, A.; Guardigli, M.; Locatelli, M.; Violante, F. S.; Rovati, L. C.; Persiani, S. Development and validation of a sensitive HPLC–ESI-MS/MS method for the direct determination of glucosamine in human plasma. *J. Chromatogr. B* **2006**, *844* (1), 119-126.
(89) Olszewski, A. J.; Szostak, W. B.; McCully, K. S. Plasma glucosamine and galactosamine in ischemic heart disease. *Atherosclerosis* **1990**, *82* (1), 75-83.
(90) Asthana, C.; Peterson, G. M.; Shastri, M. D.; Patel, R. P. Variation in the pharmacokinetics of glucosamine in healthy individuals. *Rheumatology* **2020**, *60* (3), 1205-1209.
(91) Date, J. W. The Excretion of Lactose and Some monosaccharides in the Urine in certain Pathological Conditions. *Scand. J. Clin. Lab. Invest.* **1966**, *18* (1), 45-50.
(92) Chen, Y.; Yao, Q.; Zhang, L.; Zeng, P. HPLC for simultaneous quantification of free mannose and glucose concentrations in serum: use in detection of ovarian cancer. *Front. Chem.* **2023**, *11*.
(93) Malaisse, W. J.; Sener, A.; Féry, F.; Balasse, E. O. Influence of carbohydrate intake upon plasma sorbitol concentration in normal subjects. *Am. J. Clin. Nutr.* **1981**, *34* (9), 1652-1654.
(94) Arthur, P. G.; Kent, J. C.; Potter, J. M.; Hartmann, P. E. Lactose in blood in nonpregnant, pregnant, and lactating women. *J. Pediatr. Gastroenterol. Nutr.* **1991**, *13* (3), 254-259.
(95) Campi, B.; Codini, S.; Bisoli, N.; Baldi, S.; Zucchi, R.; Ferrannini, E.; Saba, A. Quantification of d-mannose in plasma: Development and validation of a reliable and accurate HPLC-MS-MS method. *Clin. Chim. Acta* **2019**, *493*, 31-35.
(96) Fortin, E.; Ferrannini, G.; Campi, B.; Mellbin, L.; Norhammar, A.; Näsman, P.; Saba, A.; Ferrannini, E.; Rydén, L. Plasma mannose as a novel marker of myocardial infarction across different glycaemic states: a case control study. *Cardiovasc. Diabetol.* **2022**, *21* (1), 195.
(97) Panneerselvam, K.; Etchison, J. R.; Skovby, F.; Freeze, H. H. Abnormal Metabolism of Mannose in Families with Carbohydrate-Deficient Glycoprotein Syndrome Type 1. *Biochem. Mol. Med.* **1997**, *61* (2), 161-167.
(98) Etchison, J. R.; Freeze, H. H. Enzymatic assay of D-mannose in serum. *Clin Chem* **1997**, *43* (3), 533-538.
(99) Pitkänen, E.; Pitkänen, O.; Uotila, L. Enzymatic Determination of Unbound D-Mannose in Serum. *Eur. J. Chlin. Chem. Clin. Biochem.* **1997**, *35* (10), 761-766.
(100) White, L.; Ma, J.; Liang, S.; Sanchez-Espiridion, B.; Liang, D. LC–MS/MS determination of d-mannose in human serum as a potential cancer biomarker. *J. Pharm. Biomed. Anal.* **2017**, *137*, 54-59.

(101) Omosule, C. L.; Blair, C. J.; Herries, E.; Zaydman, M. A.; Farnsworth, C.; Ladenson, J.; Dietzen, D. J.; Gaut, J. P. Clinical Utility of LC-MS/MS for Blood Myo-Inositol in Patients with Acute Kidney Injury and Chronic Kidney Disease. *Clin. Chem.* **2024**, *70* (9), 1172-1181.

(102) Pouleur, A.-C.; Menghoum, N.; Cumps, J.; Marino, A.; Badii, M.; Lejeune, S.; Legault, J. T.; Boucher, G.; Gruson, D.; Roy, C.; et al. Plasma myo-inositol elevation in heart failure: clinical implications and prognostic significance. Results from the BElgian and CAnadian MEtabolomics in HFpEF (BECAME-HF) research project. *EBioMedicine* **2024**, *107*, 105264.

(103) Groenen, P. M. W.; Merkus, H. M. W. M.; Sweep, F. C. G. J.; Wevers, R. A.; Janssen, F. S. M.; Steegers-Theunissen, R. P. M. Kinetics of myo-inositol loading in women of reproductive age. *Ann. Clin. Biochem.* **2003**, *40* (1), 79-85.

(104) Quirk, J. G.; Bleasdale, J. E. Myo-inositol homeostasis in the human fetus. *Obstet. Gynecol.* **1983**, *62* (1), 41-44.

(105) Gross, M.; Reiter, S.; Zöllner, N. Metabolism of D-ribose administered continuously to healthy persons and to patients with myoadenylate deaminase deficiency. *Klin. Wochenschr.* **1989**, *67* (23), 1205-1213.

(106) Wamelink, M. M. C.; Struys, E. A.; Jansen, E. E. W.; Blom, H. J.; Vilboux, T.; Gahl, W. A.; Kömhoff, M.; Jakobs, C.; Levtchenko, E. N. Elevated concentrations of sedoheptulose in bloodspots of patients with cystinosis caused by the 57-kb deletion: Implications for diagnostics and neonatal screening. *Mol. Genet. Metab.* **2011**, *102* (3), 339-342.

(107) Rogatsky, E.; Stein, D. Novel, highly robust method of carbohydrate pre-purification by two-dimensional liquid chromatography prior to liquid chromatography/mass spectrometry or gas chromatography/mass spectrometry. *J. Chromatogr. A* **2005**, *1073* (1), 11-16.

(108) Hubinont, C.; Sener, A.; Malaisse, W. J. Sorbitol content of plasma and erythrocytes during induced short-term hyperglycemia. *Clin. Biochem.* **1981**, *14* (1), 19-20.

(109) Balint, L.; Socaciu, C.; Socaciu, A. I.; Vlad, A.; Gadalean, F.; Bob, F.; Milas, O.; Cretu, O. M.; Suteanu-Simulescu, A.; Glavan, M.; et al. Quantitative, Targeted Analysis of Gut Microbiota Derived Metabolites Provides Novel Biomarkers of Early Diabetic Kidney Disease in Type 2 Diabetes Mellitus Patients. *Biomolecules* **2023**, *13* (7), 1086.

(110) Ulaszewska, M. M.; Weinert, C. H.; Trimigno, A.; Portmann, R.; Andres Lacueva, C.; Badertscher, R.; Brennan, L.; Brunius, C.; Bub, A.; Capozzi, F.; et al. Nutrimetabolomics: An Integrative Action for Metabolomic Analyses in Human Nutritional Studies. *Mol Nutr Food Res* **2019**, *63* (1), e1800384.

(111) Petersson, G. Mass spectrometry of alditols as trimethylsilyl derivatives. *Tetrahedron* **1969**, *25* (18), 4437-4443.

(112) Wang, B.; Feng, H.; Ezeji, T.; Blaschek, H. Sugaring-Out Separation of Acetonitrile from Its Aqueous Solution. *Chem. Eng. Technol.* **2008**, *31* (12), 1869-1874.
